# Reproductive strategies of two color morphs of *Paeonia delavayi*

**DOI:** 10.3389/fpls.2025.1531186

**Published:** 2025-03-19

**Authors:** Lijun Duan, Juan Wang, Haiqing Li, Jin Li, Haizhen Tong, Chun Du, Huaibi Zhang

**Affiliations:** ^1^ College of Landscape Architecture and Horticulture Sciences, Southwest Forestry University, Kunming, China; ^2^ College of Life Science and Technology, Hubei Engineering University, Xiaogan, China; ^3^ College of Forestry, Southwest Forestry University, Kunming, China; ^4^ Institute of Forestry Industry, Yunnan, Academy of Forestry and Grassland, Kunming, China; ^5^ College of Biological and Food Engineering, Southwest Forestry University, Kunming, China; ^6^ New Zealand Institute for Plant & Food Research Limited, Palmerston North, New Zealand

**Keywords:** *Paeonia delavayi*, floral colour variation, floral colour evolution, pollinator interactions, olfactory cues, reproductive success

## Abstract

The diversity in floral coloration results from a complex reproductive system, which has evolved in response to multiple pollinators and is intricately linked to the development of pollination mechanisms. To investigate how floral trait variations influence reproduction in *Paeonia delavayi*, we conducted pollination experiments, observed insect visitation, measured floral traits, estimated petal and anther colors as perceived by pollinators and analyzed floral scent for two floral morphs (red and yellow) at two distinct sites. *P. delavayi* depended on insect pollinators for seed production. Multiple comparisons revealed that seed yields and seed sets under natural pollination were significantly higher than those under artificial pollination (homogamy and geitonogamy) and anemophilous pollination. However, there was no significant difference in seed yields(LWS, *p* = 0.487; XGLL, *p* = 0.702) and seed set (LWS, *p* = 0.077; XGLL, *p* = 0.251) between two floral morphs under natural pollination. Both morphs shared common pollinators, primarily honeybees, bumblebees, and syrphid flies. Major pollinators visited the yellow morph more frequently than the red morph, although there was no significant difference in the duration time of visits between the two morphs. Studies utilizing insect vision models, based on color reflection spectra, revealed that major pollinators could distinguish differences in petal and anther colors between the two morphs. However, there is variation in how pollinators perceive their flower colors. On the one hand, the yellow morphs contrast against the leaves background, enhancing their visual attractiveness to bees and flies. On the other hand, the red-flowered morph compensates for its visual disadvantage through olfactory cues, ensuring successful reproduction despite lower visual attractiveness. This study highlights the intricate interplay between visual and olfactory signals in plant-pollinator interactions, emphasizing their combined influence on reproductive outcomes.

## 1 Introduction

The evolution of plants is intricately linked to their pollination systems, with the combinations of pollinators for specific species varying across time and space. When pollinators switch their foraging activities among co-flowering, co-existing plant species, pollen may be transferred between different species. Interspecific pollen transfer can significantly decrease reproductive success by causing competition, influencing the separation of flowering periods or trait divergence, and leading to the deposition of heterospecific pollen on stigmas and misdirected pollen transfer during visits to flowers of different species (Wei et al., 2021). Floral fitness is optimized by improving pollen distribution to flowers of the same species and ensuring sufficient receipt of compatible pollen for ovule fertilization (Mitchell et al., 2009). Pollinator effectiveness, defined by how well a pollinator optimizes male and female fitness, is influenced by foraging patterns, floral constancy, foraging behavior, and visitation frequency (Ne’eman et al., 2010; Armbruster, 2014). These factors collectively influence the degree to which pollinators enhance plant reproductive success.

Plants might derive benefits from reduced intraspecies floral trait diversity, maintaining high floral stability. Pollinators tend to prefer visiting flowers of the same species consecutively while searching for nectars, often ignoring other rewarding flowers during the process (Gegear and Laverty, 2001). This phenomenon, known as “flower constancy,” is observed in the pollination behaviors of honeybees (Goulson, 2003; Hayes and Grüter, 2022), bumblebees (Ishii and Kadoya, 2016; Takagi and Ohashi, 2025), and dipterans (Goulson and Wright, 1998). Research indicates that pollinators possess remarkable learning abilities, enabling them to remember movement patterns or handling techniques associated with the flowers of specific species (Tsujimoto and Ishii, 2017). As a result, pollinators often remain focused on one or a few species to minimize the costs associated with re training flower-handling skills after each switch (Woodward and Laverty, 1992). Additionally, pollinators can search for flowers based on color and structure by visiting flowers of a single species or those of different species with similar colors (Wilson and Stine, 1996). Such learned behavior forms the basis for the co-evolution of plant flower colors and pollinators.

The floral traits of co-flowering plants significantly influence the degree of floral constancy. Factors such as flower color, size, structure, pollen, and nectar affect the behavior and visitation strategies of pollinators (Raguso, 2004; Kemp et al., 2019; Marquardt et al., 2021). Among these, color is a critical visual signal regulating plant–pollinator interactions (Holopainen, 2013; Chen et al., 2020b). Flower colour polymorphism refers to the variation in flower colours observed within or between natural populations of the same species, including both gradual transitions and, more prominently, discrete differences among morphs (Wang et al., 2013, 2016; Kellenberger et al., 2019; Dafni et al., 2020). The diversity of flower colors impacts the attractiveness and foraging behavior of pollinators, while pollinator-mediated selection drives changes in flower color. Guided by different flower color phenotypes, pollinators exhibit variations in visitation frequency, which may enhance gene flow between species (Marina and Brian, 1979) and contribute to the diversification of flower colors in plants (Fenster et al., 2004; Schiestl and Johnson, 2013). How do pollinators perceive and respond to changes in flower color within a population? It depends on the intensity and direction—whether facilitation or competition—of pollinator selection for flower color signals among flowering plant species (Kagawa and Takimoto, 2016; Benadi and Gegear, 2018; Whitney et al., 2020; Sapir et al., 2021; Trunschke et al., 2021).

Peony is one of the ten traditional famous flowers in China, renowned for its beauty and fragrance. *Paeonia delavayi* belonging to the family *Paeoniaceae*, genus *Paeonia*, and section *Moutan*, has been listed as a second-class nationally protected wild plant (State Forestry and Grassland Administration, Ministry of Agriculture and Rural Affairs of the People's Republic of China, 2021). It is primarily distributed in the central, northwestern, and northern regions of Yunnan Province, as well as in southeastern Tibet and western Sichuan (Zhao et al., 2021). Its flowers exhibit a rich diversity of colors, including white, pink, yellow, red, purple, and green. This diverse coloration enhances its ornamental value and makes it an important parent for breeding new varieties of cultivated peonies (Gong et al., 2003; Pan, 2015; Li et al., 2016). *Paeonia delavayi* is a cross-pollinated plant mediated by bees (Li et al., 2013). Understanding the color vision of pollinating insects in recognizing and responding to petal and anther coloration is essential for explaining pollination and reproductive efficiency differences among plants of the same species with varying color morphs. The following three scientific questions are proposed to address these aspects: (1) What are the differences in reproductive success between the two color morphs of *P. delavayi*? Is insect pollination necessary? (2) What types of pollinating insects are associated with the two color morphs of *P. delavayi*, and do they share pollinators between species? (3) Are there significant differences in floral traits between the two color morphs, and how does flower color affect visual attraction to pollinators? Pollination experiments were conducted on the two color morphs in two germplasm resource gardens to verify these questions. The impact of pollinating insects on the reproduction of *P. delavayi* was assessed, the primary pollinating insects were identified, and the pollination efficiency and visitation patterns of these insects for the two color morphs were compared. Additionally, the correlation between floral morphological characteristics and pollinators was explored. In the visual model of the primary pollinating insects, the color distances between petals and leaves and between anthers and leaf backgrounds were calculated.

## 2 Materials and methods

### 2.1 Research location and plant materials

*Paeonia delavayi* plants, established for more than 5 years, were selected as experimental materials from the Lianwang Mountain(LWS) and Shangri-La(XGLL) Germplasm Resource Garden, as well as the Shangri-La Ski Resort (**Supplementary Table 1**). Conspecifics with floral colors described as dark red, moderate red, deep red, light red, grayish red, vivid red, and deep purplish red were categorized as the red morphs, whereas conspecifics with floral colors described as vivid yellow or yellow petals with red-colored veins or spots were classified as the yellow morphs. Surveys were conducted during the flowering season from April to May of 2023 over two consecutive years.

### 2.2 Evaluation of the reproductive success of insect pollinators

The red and yellow morphs of *P. delavayi* were selected as parental plants from April to May 2022 to assess the impact of insect pollinators on plant reproduction. Six flowers of consistent size, in the late translucent stage, were chosen from each plant for six treatment groups: (1) natural pollination without any treatment (the control group), (2) emasculation without bagging to allow for natural pollination (emasculation), (3) emasculation with bagging, during which pollen was collected from the same flower and manually controlled for pollination three times during the receptive period of the stigma (artificial homogamy), (4) emasculation with bagging, during which pollen was collected from another flower of the same plant and manually controlled for pollination three times during the receptive period of the stigma (artificial geitonogamy), (5) emasculation with bagging, during which pollen was collected from a different flower of a different plant and manually controlled for pollination three times during the receptive period of the stigma (artificial xenogamy), and (6) the flowers were tagged and enclosed in nylon mesh cages (mesh size: 1 mm) to prevent insect pollination (anemophilous pollination). Between 30 and 60 flowers were allocated to each treatment. After hybridization, the flowers were tagged for identification. Mature follicles were collected according to the different experimental treatments in mid to late September, and the number of follicles, seeds, and ovules in each treatment was recorded. Seed set was calculated as: Seed set (%) = (Number of full seeds / Number of ovules per flower) × 100. One-way ANOVAs were used to evaluate differences among treatments, followed by Tukey’s tests. An independent samples t-test was conducted to assess the differences between the two morphs.

### 2.3 Survey of composition and flower-visiting frequency of insect pollinators

To clarify the differences in the composition of insect pollinators visiting the two color morphs, flowers with at least one dehisced anther (with fewer than 10 dehisced anthers) were randomly selected from different trees during the flowering period and tagged. An audio-video recorder was directed at the target flowers (daytime 8:00–18:00) to capture footage from the onset of anther dehiscence until its end, recording daily the number of dehisced anthers. Concurrently, the visiting insects were photographed using a macro camera while walking through the habitat of the target plants. Insects resting on the flowers were captured with a net, placed in bottles containing 75% alcohol, and transported to the laboratory for specimen preparation and species identification. Subsequently, the recorded videos were analyzed, and the results of the field survey were combined to document the types of flower-visiting insects, number of visits, duration of stay, and behaviors exhibited by the pollinators in the flowers (nectar feeding, pollen feeding or collecting, and predation). Floral visitors were recorded hourly and summarized the two flowering seasons dates, resulting in a total of approximately 600 observation hours for 12 trees in LWS and 950 observation hours for 19 trees in XGLL. Mann–Whitney U analysis were employed to test differences in visit frequency between the two color morphs among major pollinators. Moreover, we investigated the seed production of open flowers visited by insect pollinators in mid to late September.

A Generalized Linear Model (GLM) was employed to assess the impact of insect pollinator visitation frequency on seed production in natural pollination. First, the data were checked for normality using the Shapiro–Wilk test and transformed when needed and feasible. A multiple regression model was employed, with the seed set rate of *P. delavayi* as the response variable and the frequency of insect pollinator visits as the explanatory variable. All analyses were conducted using the “stats” package in the R programming language, and plots were generated using the “ggplot2”package.

### 2.4 Floral traits and fluorescent characteristics of anthers

To explore the potential influence of floral traits on attraction of flower-visiting insects, a total of 60 flowers from each color morph were randomly selected to measure flower diameter, flower height, stamen diameter, stamen height, and pistil height using a vernier caliper with an accuracy of 0.01 mm, and counted number of stamens and petals at their full-bloom stage. One-way ANOVAs were employed to analyze differences in flower morphology between the two color morphs. Principal Component Analysis (PCA) was conducted to detect whether there is a significant differentiation in floral traits between the two flower color morphs.

Three fully bloomed flowers from each color morph of *P. delavayi* were selected for observation. The anthers were examined and photographed using a LEICAM205 FA stereomicroscope to determine whether the epidermis of the anther wall emitted blue fluorescence under ultraviolet light. Ultraviolet images of the *P. delavayi* flowers were captured using an ultraviolet imaging device (Beijing 61, model WD-9403C, reflection wavelength 365 nm).

### 2.5 Measurement of the reflectance spectrum of flowers

One hundred and four blooming flowers and leaves were collected from LWS, and 77 blooming flowers and leaves were collected from XGLL. The samples were placed in ziplock bags and transported to the laboratory using a car refrigerator. The reflectance spectra of the petals, anthers (in the unopened state), and leaves were measured using a spectrometer (USB Ocean Optics 2000+). A fiber optic probe was installed in a black tube to minimize the influence of environmental light on reflectance spectrum measurements. A diffuse reflection whiteboard made of polytetrafluoroethylene (WS-1, Ocean Optics) was used as a reference for instrument calibration. The light source was a DH-2000 halogen-deuterium lamp (Ocean Optics Inc., Dunedin, FL). The probe was positioned at a 45° angle to the object, at a distance of approximately 5 mm, during the measurements. Measurements were taken from the most vibrant part for petals exhibiting multiple colors. Each sample was measured three times to reduce errors. Spectral data processing and color vision model simulation were conducted using the R package “pavo.” (Maia et al., 2013, 2019) Subsequent analyses, calculations, and plotting were performed in R.

### 2.6 The role of flower color in pollinator vision

Based on the findings from Section 2.3, honeybees, bumblebees, and syrphid flies were identified as the main pollinators of *P. delavayi*. In previous studies (Chen et al., 2020b; Huang et al., 2024), flower color was quantitatively analyzed within the color vision models of these pollinators.

The color hexagon model (CH model) is well suited for modeling the vision of Hymenoptera. This model represents a color in the color space as a point based on the stimulation of photoreceptors by that color. The Euclidean distance (in CH units) between color points was calculated to determine color contrast (color distance). A greater distance between the two color points indicated a higher contrast, making the colors easier to distinguish (Chittka, 1992). If the distance between the two colors was lower than 0.11 hexagonal units, bees cannot recognize them (Dyer and Chittka, 2004). A visual model was constructed using the photoreceptor sensitivity curves of *Apis mellifera* (Menzel et al., 1986; Chen et al., 2020b). Due to the lack of sensitivity curves for *Bombus (Alpigenobombus) genalis*, the curves for *Bombus terrestris*, a closely related species in the same family, were utilized to create the visual model (Skorupski et al., 2007).

A fly color vision model (the categorical color vision model) was employed to evaluate the flower colors perceived by hoverfly pollinators (Troje, 1993). This model widely applies to dipteran insects (Lunau, 2014), with a minimum color recognition distance for syrphid flies set at 0.021 Troje units (Hannah et al., 2019). Distances greater than 0.096 Troje units are easily distinguished by flies (Garcia et al., 2022), establishing 0.096 Troje units as the color recognition threshold (Huang et al., 2024).

Among these three models, the average reflectance spectrum of the corresponding leaves for the two morphs was used as the background spectrum to calculate the color contrast between petals, anthers, and their respective leaf backgrounds. A one-sample t-test was applied to assess differences in color distance between petals and leaf backgrounds and between anthers and leaf backgrounds.

### 2.7 Flower scent

The fresh petals and anthers of the two morphs were collected in LWS. and placed in a separate polyethylene-based fresh bag, and stored in a car refrigerator. Refer to Yu ‘s method (Yu et al., 2022), the flower volatiles were extracted and identified using an Agilent Technologies HP 6890 Plus Gas chromatograph (USA) (three flowers per colour morph). The GC conditions are as follows: use a DB-624UI capillary column (60m×0.32mm×1.80µm), with helium as the carrier gas, at a flow rate of 1.0 ml/min. Differences in flower scent composition between the petals and anthers of the two morphs were analyzed with permutation-based multivariate analysis of variance (PERMANOVA) using the ‘adonis’ function in the ‘vegan’ R package. The analysis was conducted using pairwise adonis (Factor: sample; Permutations = 100 000) (Anderson, 2001; Oksanen et al., 2019). The composition of flower volatiles was visualized utilizing non-metric multidimensional scaling (‘metaMDS’) according to Bray–Curtis dissimilarities. To examine the differences in the total amount of floral volatiles between the petals and anthers of the two morphs, we used a one-way ANOVA, followed by Tukey’s tests. All statistical analyses were conducted using R version 4.1.1.

## 3 Results

### 3.1 Reproductive success of insect pollinators

Significant differences in seed yields (the number of seeds per flower) (one-way ANOVA, the red morphs, F_5,234_ = 20.11, *p* < 0.001; the yellow morphs, F_5,234_ = 27.14, *p* < 0.001) and seed sets (one-way ANOVA, the red morphs, F_5,234_ = 22.97, *p* < 0.001; the yellow morphs, F_5,234_ = 33.18, *p* < 0.001) were observed across different pollination methods in LWS. Multiple comparisons revealed (**Table 1**) that seed yields and seed sets under natural pollination were significantly higher than those under artificial pollination (homogamy and geitonogamy), anemophilous pollination, and emasculation without pollination (*p* < 0.05, Tukey test). However, seed yields (*p* = 0.206, Tukey test) and seed sets (*p* = 0.455, Tukey test) under artificial xenogamy from different individuals of the yellow morphs did not differ significantly from those under natural pollination, with no significant difference in seed yields (one-sample *t*-test, *t* = − 0.697, d.f. = 118, *p* = 0.487) and seed sets (one-sample *t*-test, *t* = −1.785, d.f. = 118, *p* = 0.077) between yellow-flowered and red morphs under natural pollination.

**Table 1:** Effects of different pollination treatments (mean ± s.e.) among the two morphs of *P. delavayi* in LWS.

Parameters	Morphs	Treatment								
		Natural pollination	Emasculation	Artificial homogamy	Artificial geitonogamy	Artificial xenogamy	Anemophilous pollination	F	df	P
		(n = 60)	(n = 40)	(n = 40)	(n = 40)	(n = 20)	(n = 40)			
Seed yields	Red	7.83 ± 0.71a	3.00 ± 0.54b	1.13 ± 0.37c	4.18 ± 0.73b	4.6 ± 1.54b	0.33 ± 0.1c	20.11	5	< 0.001
	Yellow	7.95 ± 0.57a	5.23 ± 0.64b	1.56 ± 0.46cd	3.77 ± 0.65bc	5.8 ± 0.88ab	0.38 ± 0.12d	27.14	5	< 0.001
Fruit set (%)	Red	41.71 ± 3.14a	19.11 ± 3.24b	6.95 ± 2.5c	24.55 ± 3.84b	20.56 ± 5.82b	2.32 ± 0.93c	22.97	5	< 0.001
	Yellow	49.28 ± 2.84a	35.91 ± 3.56b	8.73 ± 2.39c	25.83 ± 4.36b	39.52 ± 5.36ab	3.39 ± 1.43c	33.18	5	< 0.001

Values (mean ± s.e.) with different letters in the same row indicate significant differences between treatments according to Tukey's tests at p < 0.05.

Due to the limited number of yellow-flowered *P. delavayi* in XGLL, only red morphs were used for the pollination experiment. The variation in seed yields (one-way ANOVA, F_5,154_ = 5.448, *p* < 0.001) and seed sets (F_5,154_ = 5.328, *p* < 0.001) differed significantly among treatments. Multiple comparisons indicated (**Supplementary Table 2**) that seed yields and seed sets for natural pollination were higher than for artificial pollination (homogamy, geitonogamy) and anemophilous pollination (*p* < 0.05, Tukey test). However, seed yields and seed sets in artificial xenogamy were slightly greater than those in natural pollination, with no significant difference between the two methods (*p* > 0.05, Tukey test). In addition, there was no significant difference in seed yields (one-sample *t*-test, *t* = − 0.385, d.f. = 44, *p* = 0.702) and seed sets (one-sample t-test, t = − 1.163, d.f. = 44, p = 0.251) of natural pollination between the two morphs.

### 3.2 Insect pollinators and visitation frequency

**Figure 1:**
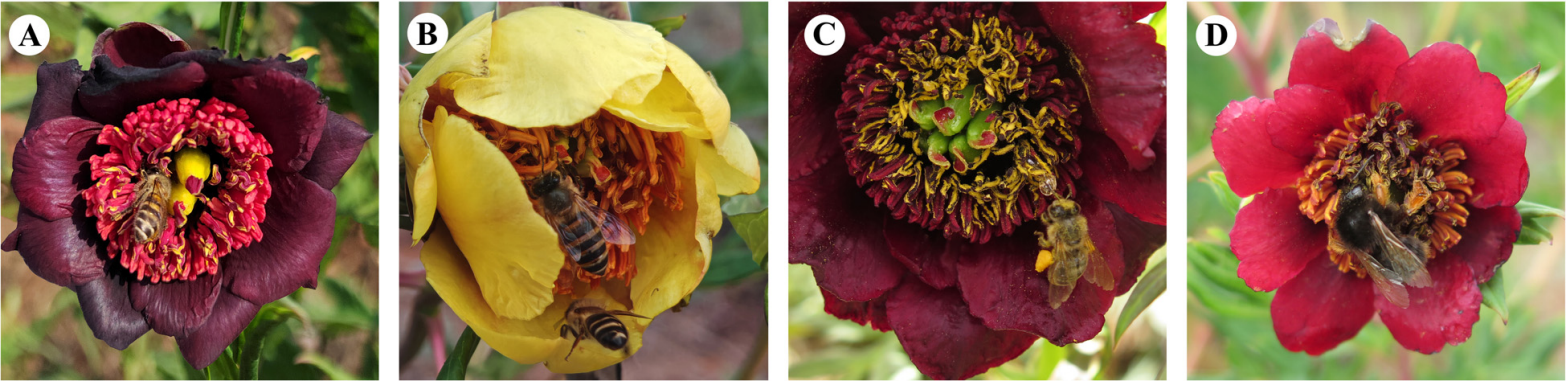
Insects visiting *P. delavayi* flowers. **(A, B)** Honeybee visiting a red and yellow flower; **(C)** Honeybee visiting a nectar-bearing flower. **(D)** Bumblebee (*B.* (*Alpigenobombus*) *genalis*) visiting a red flower;.

Nine species of flower-visiting insects were associated with *P. delavayi* in LWS, belonging to three orders and six families (**Supplementary Table 3**). Hymenoptera were the most numerous, with five species present. They were Halictidae sp., Apis sp., *B. (Alpigenobombus) genalis*, *Formica fusca* Linnaeus, and *Formica sinensis* Wheeler. *F. fusca* and *F. sinensis* remained on the flowers for extended periods to feed on nectar but did not promote pollen transfer. Halictidae sp. had an exceptionally low flower-visiting frequency, primarily stealing pollen and nectar. Apis sp. and *B. (Alpigenobombus) genalis* actively collected pollen from the stamens or fed on nectar by flying or crawling. Only one species of Diptera, Syrphidae sp., stayed on the flowers for a long time, feeding on nectar and licking pollen. Three species of Hemiptera, *Lygaeus vicarius*, *Dysdercus* sp., and *Nysius ericae*, did not interact with the anthers or stigma during their visits and exhibited low visitation frequencies. According to pollinator standards (Huang et al., 2024), the primary pollinating insects were honeybees (**Figures 1A–C**), bumblebees (**Figure 1D**), and syrphid flies. Mann–Whitney U test revealed that there was no significant difference in the residence time (per flower per hour) of flower visits for honeybees (*U* = 341, n_1_ = 30, n_2_ = 30, *p* = 0.105), bumblebees (*U* = 323, n_1_ = 30, n_2_ = 30, *p* = 0.052), and syrphid flies (*U* = 353, n_1_ = 30, n_2_ = 30, *p* = 0.135) between the two morphs. However, the number of visits by honeybees (*U* = 311, n_1_ = 30, n_2_ = 30, *p* = 0.040), bumblebees(*U* = 324, n_1_ = 30, n_2_ = 30, *p* = 0.045), and syrphid flies(*U* = 313, n_1_ = 30, n_2_ = 30, *p* = 0.034) to the yellow morphs were significantly higher than those of the red morphs (**Table 2**).

**Table 2:** Visitation frequency and residence time(s) (mean ± s.e.) by major pollinators of *P. delavayi* in LWS.

morphs	Insect pollinator	*Apis* sp.	*Bombus (Alpigenobombus) genalis*	*Syrphidae* sp*.*
Red Morph	Visitation frequency (visits/flower/h)	2.17 ± 0.36^a^	0.41 ± 0.12^a^	0.11 ± 0.02^a^
Yellow Morph		4.36 ± 0.72^b^	1.32 ± 0.36^b^	0.30 ± 0.06^b^
Red Morph	Residence time (s/flower/h)	89.37 ± 20.14^a^	28.62 ± 7.96^a^	20.53 ± 6.62^a^
Yellow Morph		176.17 ± 36.41^a^	55.74 ± 12.19^a^	57.54 ± 16^a^

Values (mean ± s.e.) with different letters in the same column indicate significant differences according to Mann–Whitney U tests at p < 0.05.

Seven species of insects visited the flowers of *P. delavayi* in XGLL, also belonging to three orders and six families (**Supplementary Table 3**). *Lasius himalayanus* Bingham, *Musca domestica*, *Lygaeus vicarious*, *Dysdercus* sp., and *N. ericae* primarily fed on nectar or pollen without contacting the anthers or stigma. At the same time, Apis sp. and Syrphidae sp. facilitated pollen transfer. According to pollinator standards (Huang et al., 2024), the primary pollinating insects were honeybees and syrphid flies. There was no significant difference in the number of visits (*U* = 870, n_1_ = 60, n_2_ = 35, *p* = 0.163) and duration (per flower per hour) (*U* = 855, n_1_ = 60, n_2_ = 35, *p* = 0.131) for honeybees between the two morphs. Notably, syrphid flies had longer visits(*U* = 675, n_1_ = 60, n_2_ = 30, *p* = 0.049) and residence time (*U* = 663, n_1_ = 60, n_2_ = 30, *p* = 0.040) to the yellow morphs compared to the red morphs (**Supplementary Table 4**).

#### 3.2.1 Visitation frequency of primary pollinating insects and daily activity patterns

The daily activity patterns of different pollinating insects exhibited distinct characteristics. In LWS, honeybees demonstrated two peak visitation periods: 10:00 to 11:00 and 13:00 to 14:00 (**Supplementary Figure 1A**). bumblebees peaked between 11:00 and 12:00 (**Supplementary Figure 1E**), while Syrphid flies peaked from 12:00 to 13:00 (**Supplementary Figure 1C**). In XGLL, honeybees peaked from 11:00 to 12:00 and 14:00 to 15:00 (**Supplementary Figure 1B**), while syrphid flies peaked from 12:00 to 13:00 (**Supplementary Figure 1D**). Overall, the peaked activity periods of pollinating insects were staggered, reducing competition for the pollen of *P. delavayi* and enhancing complementary pollination. The main daily activity patterns of pollinating insects showed similar trends across both color morphs.

#### 3.2.2 Visitation frequency of primary pollinating insects at different pollen dispersal speeds by stamen

**Figure 2:**
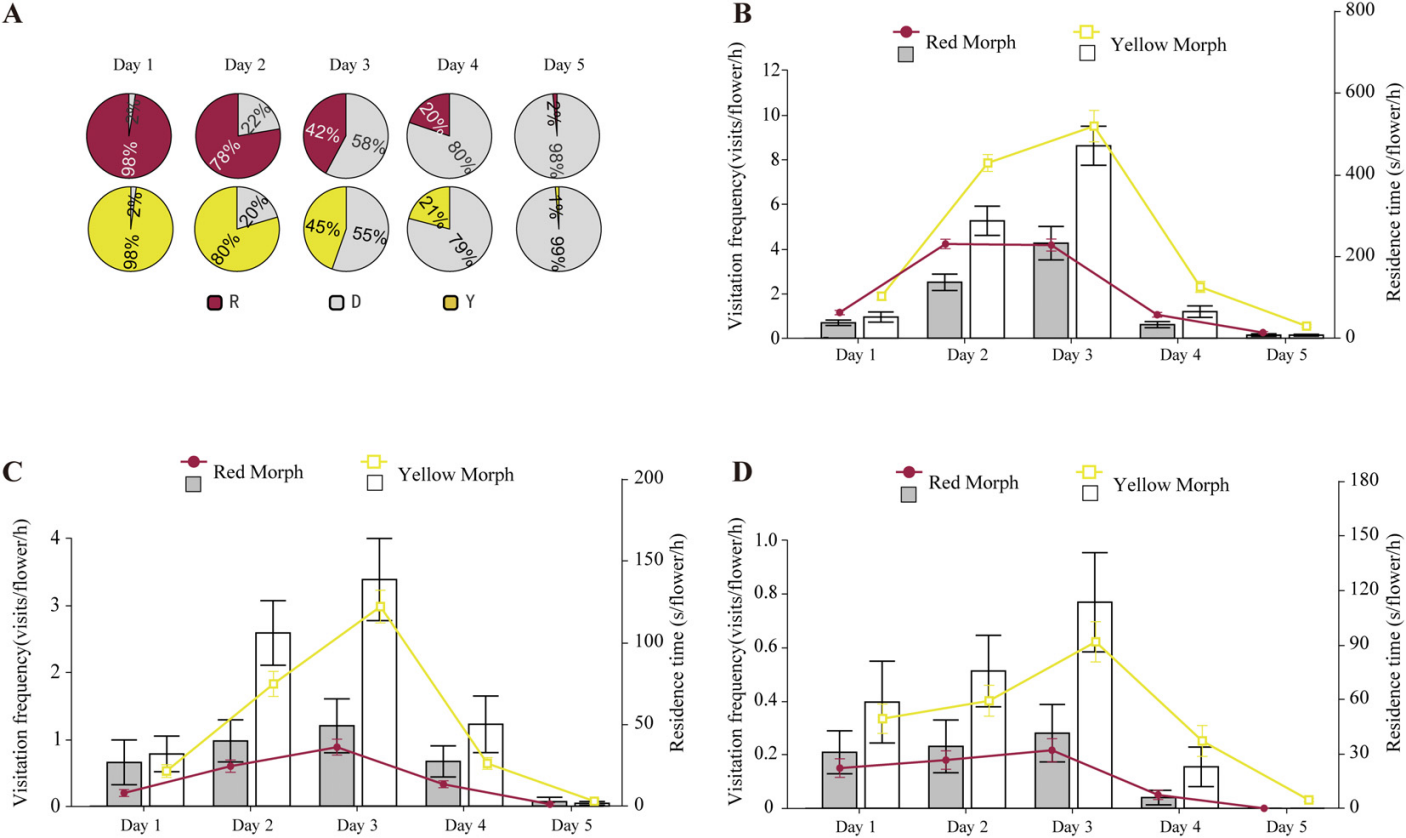
Visitation frequency and residence time(s) (mean ± s.e.) of honeybees **(B)** and bumblebees **(C)** and syrphid flies **(D)** in LWS, across different anther dehiscence schedules **(A)**. R represents red indehiscent anthers, Y represents yellow indehiscent anthers, and D represents dehiscent anthers.

The gradual pollen presentation strategy of *P. delavayi* facilitated distribution to more pollinators. The visitation frequency of honeybees correlated with the amount of pollen exposed in the anthers. Statistics indicated that when the petals were in the initial blooming stage, and the stamens had not yet cracked to release pollen, honeybees rarely lingered on the flowers; if they did visit, they left quickly. As the amount of pollen released from the cracking anthers increased, honeybee visitation visits initially rose and then declined. In LWS, the visitation frequency of honeybees, bumblebees and syrphid flies reached its peak on the third day of pollen release when 55%–58% of the stamens were cracked. However, as the number of cracked stamens increased from 79% to 80%, the visitation frequency gradually decreased, falling to nearly zero when 98% of the stamens were cracked(**Figures 2A–D**). In XGLL, the highest frequency of honeybee visitation visits occurred when 48% to 54% of the stamens were cracked (**Supplementary Figures 2A, B**). The visitation patterns of syrphid flies (**Supplementary Figure 2C**) mirrored those of honeybees. Across different pollen release rates, the primary pollinating insects exhibited similar trends in both color morphs.

### 3.3 Effects of insect visitation frequency on seed set

**Figure 3:**
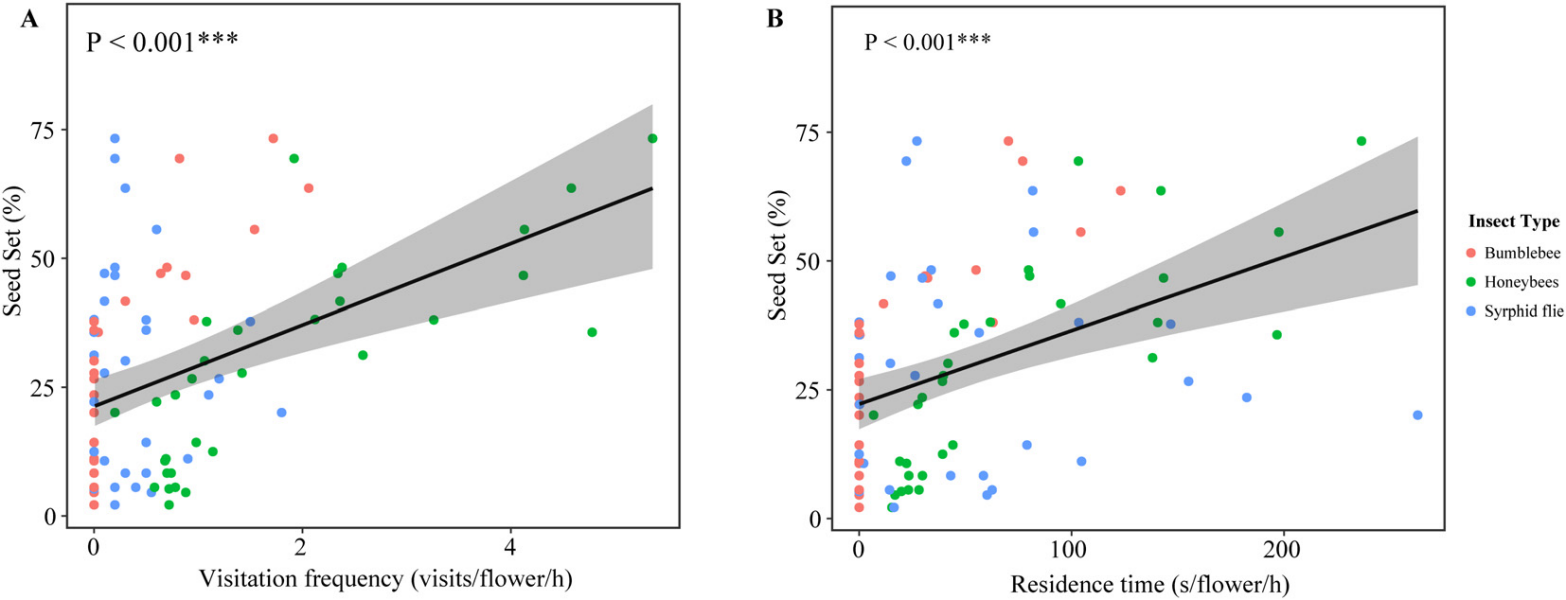
Results of linear regression analysis testing the response of the seed set of *P. delavayi* in each flower in average frequency **(A)** and residence time **(B)** of insect visits. Linear regressions ± 95% confidence intervals are depicted. ***indicate significant difference at 0.001 level.

The generalized linear models demonstrated that the effect on seed production in natural pollination showed a positive effect of the visitation frequency of honeybees (GLM, *p* = 0.0147) and bumblebee (GLM, *p* = 0.0303) (**Supplementary Table 5**). On the other hand, the residence time of honeybees (GLM, *p* = 0.00198) and bumblebee(GLM, *p* = 0.00563) in each flower positively affected seed production (**Supplementary Table 6**). However, the seed set was not associated with the visitation frequency(GLM, *p* = 0.3491) and the residence time(GLM, *p* = 0.59337) of syrphid flie. Overall, the seed set was positively affected by insect visit frequency (R^2^ = 0.6657, F-statistic=20.91, d.f. = 3,27, *p* < 0.001; **Figure 3A**) and the residence time (R^2^ = 0.6916, F-statistic=23.42, d.f. = 3,27, *p* < 0.001; **Figure 3B**). These results demonstrated that higher pollinator visitation rates resulted in a higher seed set at two distinct sites.

### 3.4 Floral traits and fluorescence characteristics of anthers

There were significant differences in flower diameter, flower height, stamen diameter, stamen height, pistil height, stamen-pistil shortest distance, number of stamens, and petal number of the two morphs in two distinct elevational sites. Specifically, number of stamen of the red morphs was significantly larger than those of the yellow ones (**Supplementary Table 7**), implying that they provided more pollen for pollinators. The spatial separation between the anthers and stigmas in plants of the two color morphs limited self-pollination within the same inflorescence, necessitating assistance from wind or other insects. Principal component analysis (PCA) results indicated that the first principal coordinate component explained 29.8% to 38.7% of the variation in floral traits, while the second principal coordinate component explained 22.3% to 23.9% of the variation. It was evident that there was a significant overlap in floral traits between individuals of the two color morphs (**Supplementary Figures 3A, B**).

**Figure 4:**
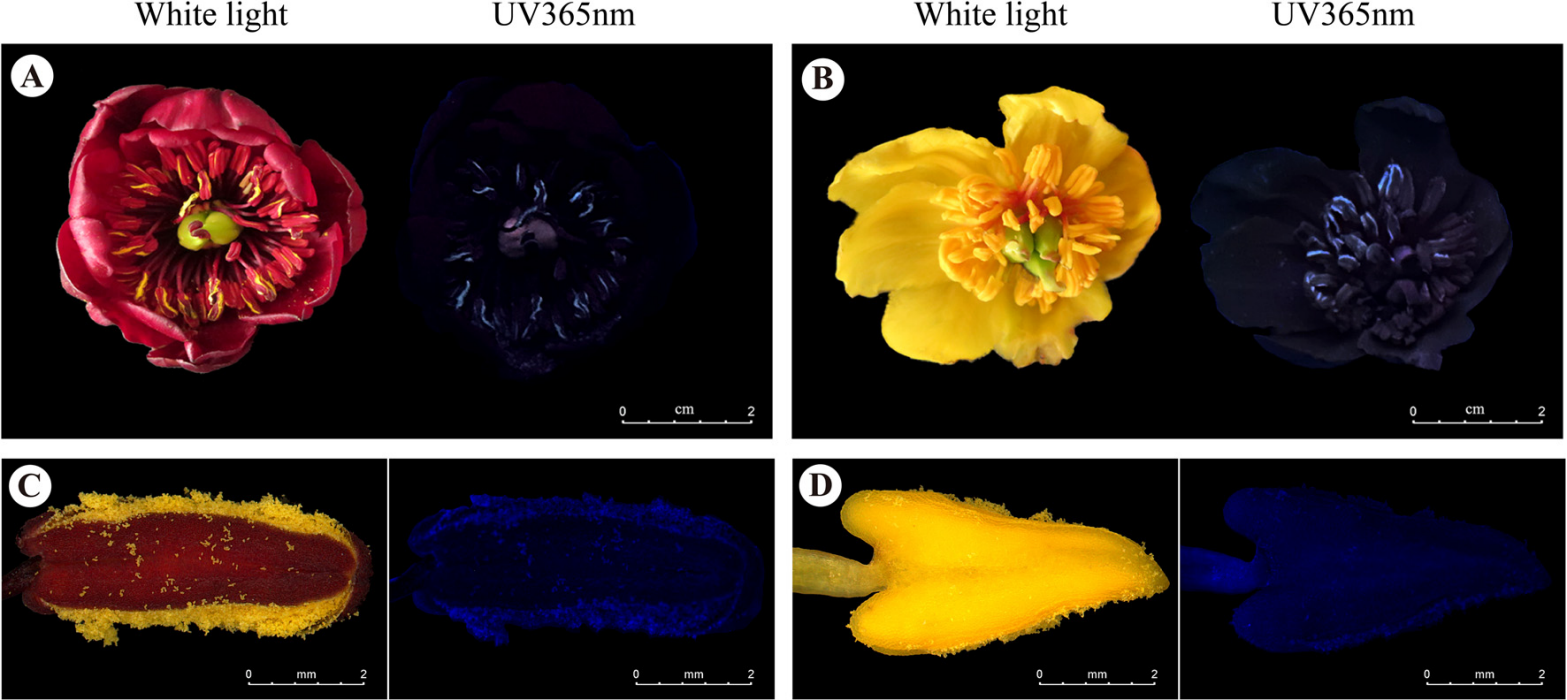
Photographs of the flowers and anthers in LWS. **(A, B)** Flower of *P. delavayi* and **(C, D)** its anther under white light (left) and UV 365 nm light (right). Scale bars, 2 mm for **A–D**.

When the anthers of red-flowered and yellow morphs released pollen, they emitted different fluorescence under 365 nm ultraviolet light (**Figures 4A–D**; **Supplementary Figures 4A–D**). In contrast, the petals did not exhibit fluorescence at this wavelength. The pollen in the stamens provided visual cues for honeybees from a distance.

### 3.5 Main flower colors perceived by primary pollinators

**Figure 5:**
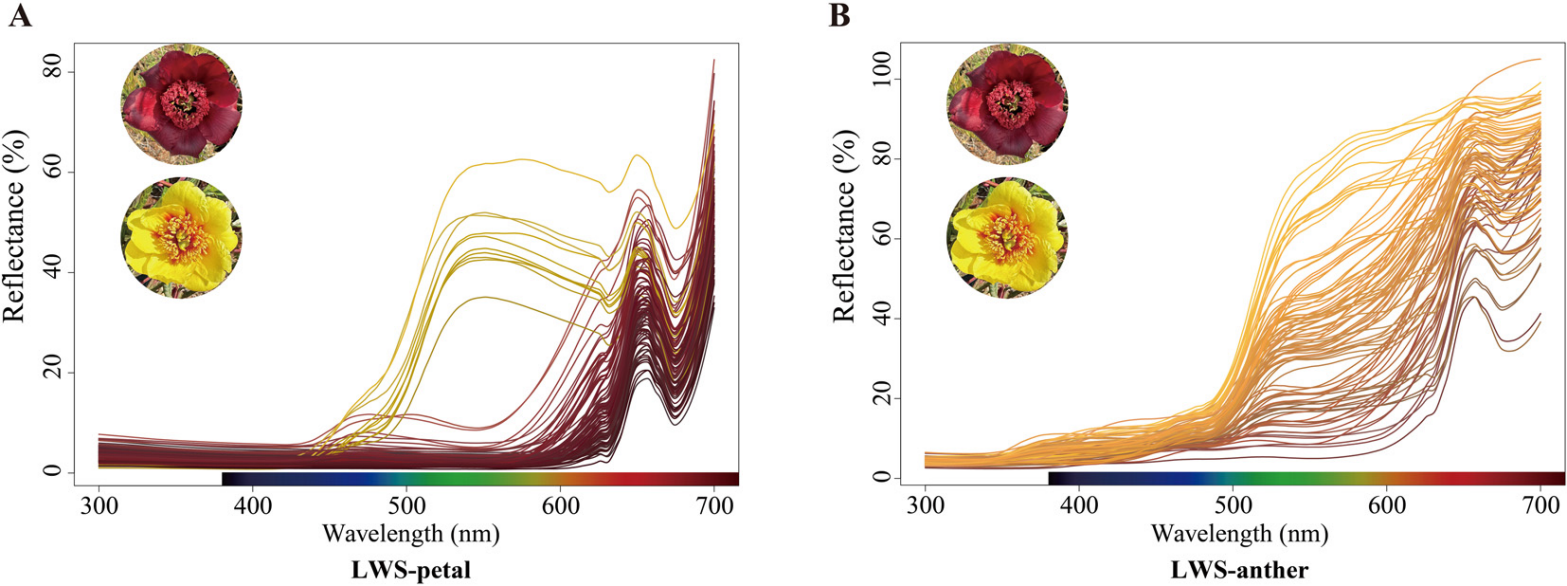
Diffuse reflectance spectra of *P. delavayi* flowers. **(A)** Reflectances of petals and **(B)** its anthers in LWS.

The reflection spectra of the red-flowered and the yellow morphs in distinct elevational sites were measured, revealing that the middle petals and anthers of both morphs exhibited similar absorption values at ultraviolet (300–400 nm) and red wavelengths (600-700 nm), and had an absorption peak around 650 nm. However, the yellow morphs showed a significantly greater absorption value than the red ones at green wavelengths (500–600 nm) (**Figures 5A, B**; **Supplementary Figures 5A, B**).

**Figure 6:**
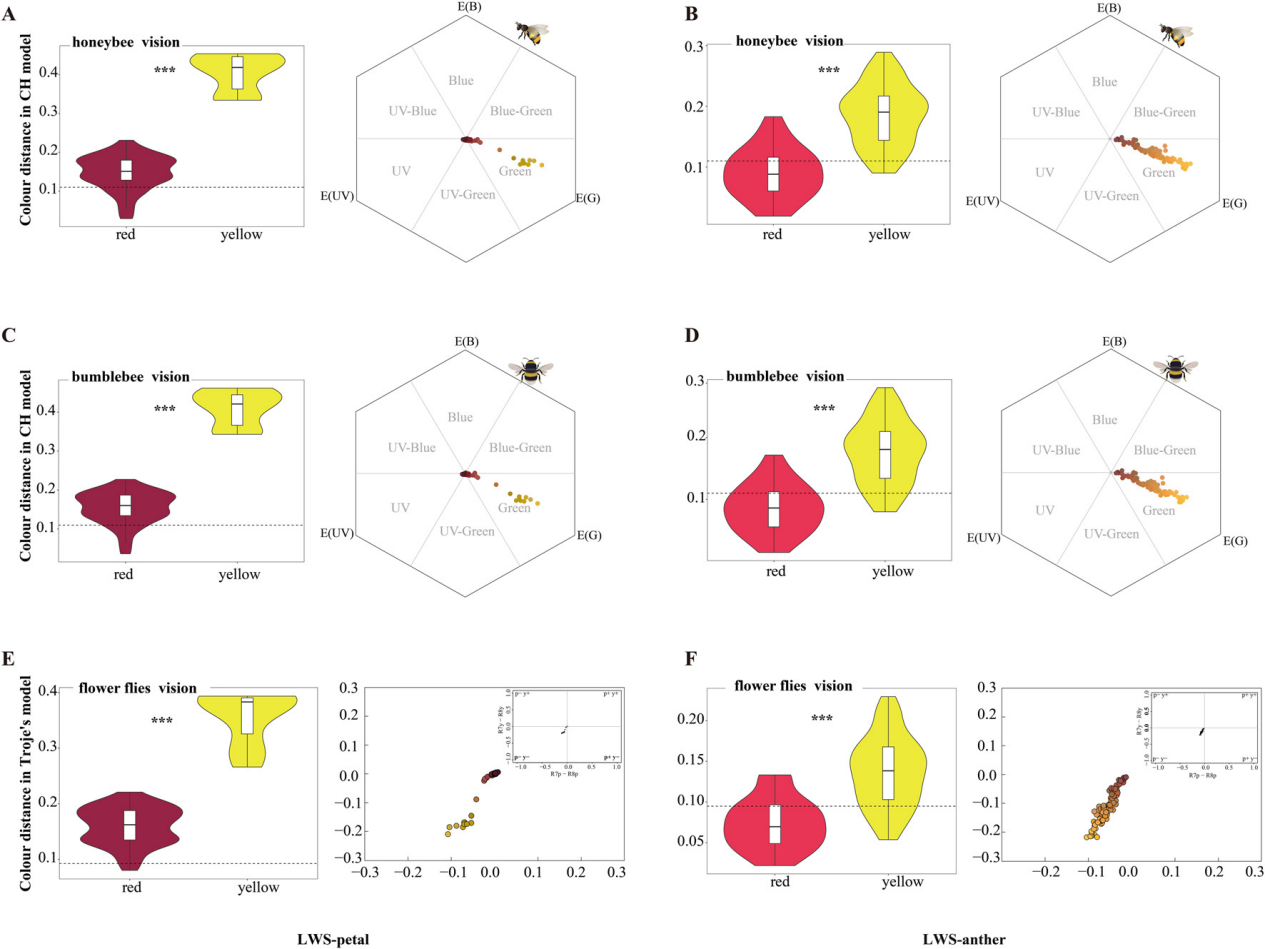
Flower characteristics and color loci in pollinator color spaces in LWS. **(A, B)** Colour distances between petals and leaves, as well as anthers and leaves, calculated using the bee color hexagon model. The dashed line indicates 0.11 hexagon units, which is the discrimination threshold of bees. **(C, D)** Color distances between petals and leaves, as well as anthers and leaves, calculated using the bumble bee color hexagon model. The dashed line indicates 0.11 hexagon units, which is the discrimination threshold of bumblebees. **(E, F)** Color distances between petals and leaves, as well as anthers and leaves, calculated in the fly color model. The dashed line indicates the fly threshold of 0.096 Troje units. ***p < 0.001.

Significant differences in color distances were observed for the petals and anthers of the various colored flowers in the honeybee visual model (hexagon model) (one-way ANOVA, *F*_7,342_ = 48.180, *p* < 0.001). The mean chromatic contrast between yellow and red petals and background leaves varied(LWS and XGLL) significantly exceeded 0.11 hexagon units (one-sample *t*-test, *t* = 10.820, d.f. = 180, *p* < 0.001; **Figure 6A**; **Supplementary Figure 6A**), as did the contrast between yellow anthers (LWS and XGLL) and red anthers (XGLL) (one-sample *t*-test, *t* = 10.366, d.f. = 142, *p* < 0.001; **Figure 6B**; **Supplementary Figure 6B**). This finding suggested that honeybee pollinators could effectively distinguish petals and anthers from the leaf background. By contrast, the mean chromatic contrast of red anthers and background leaves(LWS) (mean colour distance = 0.090 CH units) was significantly lower than the threshold(one-sample *t*-test, *t* = −2.413, d.f. = 25, *p* < 0.05; **Figure 6B**), indicating that they were unable to distinguish red anthers from the leaf background.

Notable differences in color distances were observed for the petals and anthers of the different colored flowers in the bumblebees visual model (one-way ANOVA, *F*_7,342_ = 45.821, *p* < 0.001). The average chromatic contrast between yellow and red petals and background leaves varied (LWS and XGLL) significantly exceeded 0.11 hexagon units (one-sample *t*-test, *t* = 11.708, d.f. = 180, *p* < 0.001; **Figure 6C**; **Supplementary Figure 6C**), as did the contrast between yellow anthers (LWS and XGLL) and red anthers (XGLL) (one-sample *t*-test, *t* = 9.441, d.f. = 142, *p* < 0.001; **Figure 6D**; **Supplementary Figure 6D**). This result indicated that bumblebee pollinators could effectively distinguish petals and anthers from the leaves background. However, the average chromatic contrast of red anthers and background leaves varied (LWS) was only 0.085 CH units, falling below the recognition threshold for bumblebees (one-sample *t*-test, *t* = −3.204, d.f. = 25, *p* < 0.05; **Figure 6D**), suggesting that they could not distinguish red anthers from the leaf background.

Marked variations in color distances were observed for the petals and anthers of the different colored flowers in the fly visual model (one-way ANOVA, *F*_7,342_ = 58.730, *p* < 0.001). The average chromatic contrast between yellow and red petals and background leaves varied (LWS and XGLL) significantly exceeded 0.096 Troje units (one-sample *t*-test, *t* = 19.854, d.f. = 180, *p* < 0.001, **Figure 6E**; **Supplementary Figure 6E**), as did the contrast between yellow anthers (LWS and XGLL) and red anthers (XGLL) (one-sample *t*-test, *t* = 10.331, d.f. = 142, *p* < 0.001; **Figure 6F**; **Supplementary Figure 6F**). This result indicated that fly pollinators could easily distinguish petals and anthers from the leaves background. By contrast, the average chromatic contrast of red anthers and background leaves(LWS) (mean colour distance = 0.073 Troje units) was notably below the threshold(one-sample *t*-test, *t* = −3.690, d.f. = 25, *p* < 0.05; **Figure 6F**), implying that they could not accurately discriminate red anthers from the leaf background.

### 3.6 Flower scent

**Figure 7:**
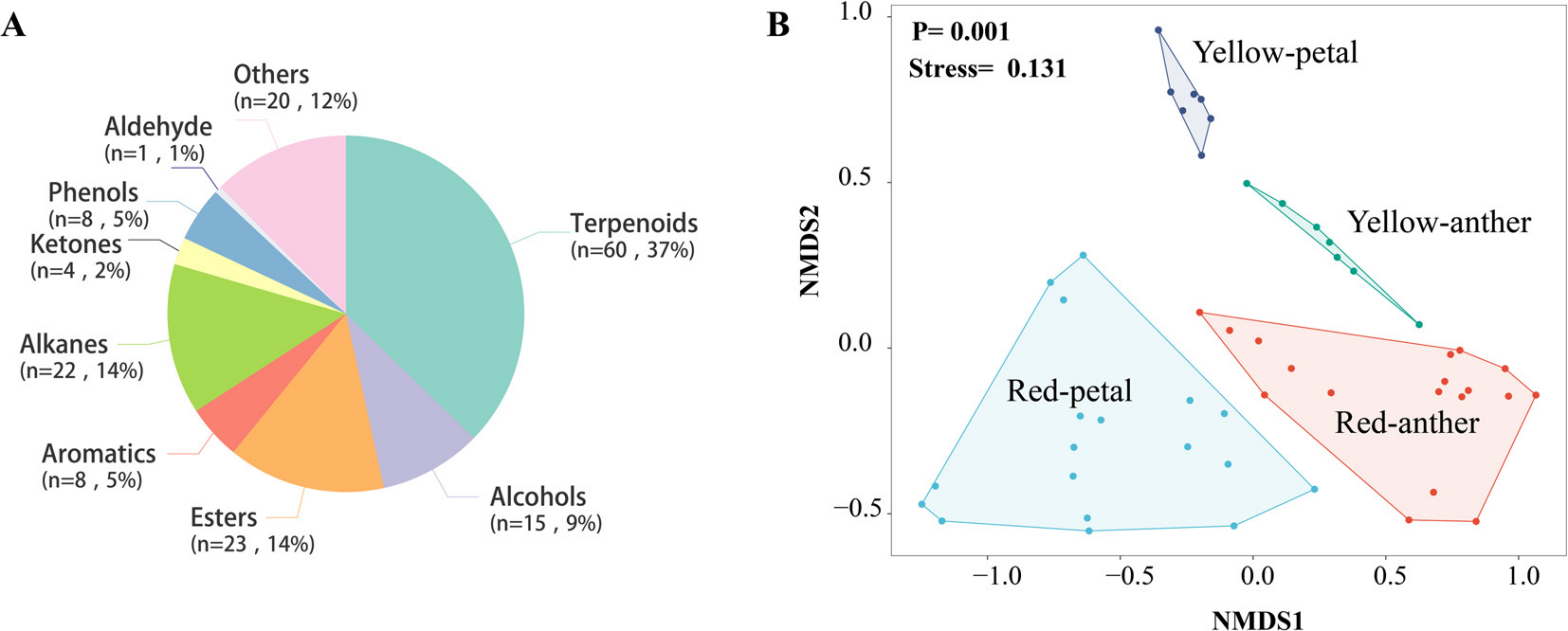
Floral volatile composition **(A)** and differences in both the petals and anthers of the two morphs in LWS, analyzed using non-metric multidimensional scaling **(B)**.

Research on the chemical composition of *P. delavayi* flowers reveals that a total of 161 compounds were detected in both the petals and anthers of the two morphs in LWS (**Supplementary Table 8**). Among the identified compounds, terpenoids were the most abundant (60 compounds, 37%), followed by alcohols (15 compounds, 9%), esters (23 compounds, 14%), aromatics (8 compounds, 5%), alkanes (22 compounds, 14%), ketones (4 compounds, 2%), phenols (5 compounds, 5%) and others compounds (20 compounds, 12%) (**Figure 7A**).

Petals of the red morphs contained a greater diversity of terpenes of petals (43 compounds) and anthers (29 compounds) than the yellow ones (petals, 25 compounds; anthers, 20 compounds). The most abundant terpenes in the red morphs were caryophyllene, terpinolene, α-copaene, β-cubebene, β-cadinene, β-funebrene, DL-limonene and linalool. Notably, the content of linalool was considerably higher in both the petals(15.02 ± 0.31) and anthers (4.3 ± 0.18) of red-flowered plants than yellow-flowered ones (petals,0.79 ± 0.28; anthers, 0.14 ± 0.03).

The total concentration of floral volatiles did not differ significantly between the petals and anthers of the two morphs (one-way ANOVA; F_3,46_ = 0.464, *p* = 0.709). However, the composition of floral scents differed significantly between the petals and anthers of the two morphs (PERMANOVA, F_3,46_ = 4.684, R^2^ = 0.56, *p* = 0.001) (**Figure 7B**). Overall, the the two morphs exhibited distinct volatile compositions.

## 4 Discussion

### 4.1 Breeding system evaluation and outcrossing rate

The breeding system is a crucial factor influencing the genetic diversity of plants (Ollerton et al., 2011). This study conducted extensive field pollination experiments on *P. delavayi*, confirming it as a cross-pollinated species reliant on pollinators. Both red and yellow morphs could produce seeds through artificial self-pollination, although the seed yields and sets rates were low. Notably, seed yields and sets rates under artificial monogamy pollination were significantly lower than those under artificial cross-pollination between different plants. However, there were no significant differences between artificial cross-pollination and natural pollination. These findings suggest that under natural conditions, *P. delavayi* could achieve effective cross-pollination via pollinating insects. In Shangri-La, seed set from artificial cross-pollination was marginally higher than that from natural pollination in both morphs, suggesting potential pollen or pollinator limitation (Sorokhaibam et al., 2024). Seed sets under natural and artificial pollination in *P. delavayi* below 50%. Similarly, other species in the genus Paeonia also exhibit low seed sets (Luo et al., 1988; Li et al., 2013; Peter et al., 2013; Chen et al., 2023). These low seed sets could contribute significantly to the endangerment of plants within the genus Paeonia.

Several ecological factors could potentially influence seed set in naturally pollinated plants. Firstly, species with analogous floral features and with similar blooming periods may contend for the service of the same pollinators, which could lead to lowered reproductive success (Mesgaran et al., 2017). In this study, we noted several co-flowering plants with significant abundances, such as *Paeonia suffruticosa* and *Paeonia lactiflora*, which likely competed for pollinators, negatively impacting the reproductive success of *P. delavayi*. Moreover, the abundance and geographic range of *P. delavayi* have been notably influenced by human-induced disturbances, leading to habitat fragmentation (Chen and Zuo, 2019). This fragmentation not only alters pollinator diversity but also influences other factors that affect seed set, such as the size of habitat patches and the density of flowering plants (Conner and Rush, 1996). Additionally, *P. delavayi*’s reliance on less efficient pollinators may result in substantial declines in pollination success, particularly if harsh environmental conditions and climate change disrupt pollinator activity (Cosacov et al., 2008). In certain populations, flowers that were pollinated manually yielded a lower number of seeds compared to those pollinated naturally. This difference might be potentially due to stigma harm resulting from hand pollination, adverse impacts of excessive pollen concentration on pollen tube growth (stigma clogging), lower diversity of pollen donors, and pollen transfer by insect pollinators treating the stigma as a food source. Additionally, the delivery of inadequate or incompatible pollen during hand pollination may contribute to the observed lower seed set (Young and Young, 1992). We propose that conservation strategies for plant species with low seed set might be improved by promoting pollinator visits and movement between individuals of the same species. These goals can be accomplished by strengthening the competitive edge of plant populations (Mayer et al., 2012), improving the management of surrounding habitats to enhance pollinator activity (Ghazoul, 2006), and decreasing the spatial separation between conspecific populations to foster greater gene flow (Van Rossum and Triest, 2010).

### 4.2 Shared pollinator mediation

When plants in the same area share pollinators, pollinator-mediated competition can enhance trait diversity (Bergamo et al., 2018). For instance, studies on hummingbird–plant interactions in South American temperate forests reveal that interspecies competition driven by shared pollinators leads to diversifying plant traits (Aizen and Rovere, 2010). Research in the Andes has shown that Solanaceae can evolve new flower colors due to such competitive dynamics (Muchhala and Thomson, 2012). Pollinator-mediated characteristics of the same species across different regions may vary based on pollinator types and preferences. For example, *Papaver rhoeas* in its native Eastern Mediterranean is primarily pollinated by local beetles attracted to visible red light. At the same time, poppies introduced to Central Europe have adapted to reflect red and ultraviolet light to attract honeybees (Martinez-Harms et al., 2020). Bees were the major pollinators during the flowering period of *Paeonia* plants (Li et al., 2013; Peter et al., 2013), and are found to be effective pollinators in areas where they are the predominant species of wild hymenopterans (Sorokhaibam et al., 2024). Syrphid flies played an important role in plant pollination in high-altitude ecosystems (McCabe and Cobb, 2021). Our results showed that the two morphs of P. *delavayi* share common pollinators at the same site but exhibit different color preferences, as evidenced by varying visitation frequencies, which may influence floral color divergence patterns. At Liangwang Mountain, honeybees, bumblebees, and syrphid flies visited the yellow morphs more frequently than the red morphs. Notably, in Shangri-La, syrphid flies not only visited the yellow morphs more often but also spent longer durations on them compared to the red morphs. Geographic differences concerning floral visitor preferences can stem from variations in pollinator assemblage, frequency, and variability (Price et al., 2005). As a result, foraging preferences influenced by pollinators may cause shifts in the optimal floral traits (Gómez et al., 2009). This suggests that pollinators may facilitate gene flow among plants of the same species in a shared environment (Vazquez et al., 2005; Landry, 2013).

Pink is likely the ancestral flower color of Paeonia plants, evolving into other colors with pink components as well as white, while yellow is considered a more derived flower color (Yuan and Wang, 2003). Floral color evolution in *P. delavayi* is primarily driven by the pollinator-driven selective influences that have pronounced preferences for specific traits (Kagawa and Takimoto, 2016; Chen and Zuo, 2019; Sapir et al., 2021; Trunschke et al., 2021). In this study, two color morphs of *P. delavayi* display relatively large interspecific variations in flower color. Over two years of observation, honeybees were found to rarely switch between the two morphs within the same study region. Additionally, the handling skills of pollinators influence their visiting behavior. For example, in blue-white and blue-yellow bicolored artificial flower clusters, honeybees exhibit color constancy, favoring either blue or yellow flowers. When rewards are provided, pollinators are not restricted by flower color to visit shallow-well flowers (Sanderson et al., 2006). The red morphs compensate for vision differences by increasing pollen production and adjusting the schedule of pollen presentation to match the abundance and efficiency of pollen transfer by their pollinators.

### 4.3 Sensory characteristics of pollinators and reproductive success

Plants convey pollination signals through flower colors to attract pollinators (Kemp et al., 2019). Hymenoptera, such as bees and bumblebees, possess trichromatic colour vision with maximum sensitivities in the ultraviolet(λmax ≈ 340 nm), blue(λmax ≈ 430 nm) and green (λmax ≈ 535 nm) regions (Peitsch et al., 1992; Zhang et al., 2024). However, they struggle to distinguish visible red signals because they lack red photoreceptors in their eyes (Dyer et al., 2021). Pure red flowers provide a moderate stimulation to green receptors at the edge of pollinators’ spectral sensitivity. This stimulus, combined with moderate stimulation of the ultraviolet and blue receptors, produces equal signals across all receptors, leading to an achromatic perception (Chittka, 1992; Chittka and Waser, 1997). Such flowers may still be detected and discriminated by honeybees and bumblebees through achromatic contrast (Martínez-Harms et al., 2010), although this mechanism is relatively weak (Lunau et al., 2011). Furthermore, although rare, some red flowers in nature are indeed pollinated by bees (McNaughton and Harper, 1960; Chittka et al., 1994; Chittka and Waser, 1997; Martínez-Harms et al., 2010; Chen et al., 2020a), rather than all red flowers being exclusively bird-pollinated. Our results showed that the mean color distance between petals and leaves of red-flowered *P. delavayi* is slightly above the discrimination threshold of the relevant model, indicating that the red morphs are still detectable by bees (Chittka and Waser, 1997). However, to honeybees, the red morphs appear less conspicuous against the leaf background compared to yellow morphs. Nevertheless, this plant provides abundant nectar (Li et al., 2013, 2023) and pollen rewards to pollinators, which attract pollinators visits. This may explain why honeybees visit both the red and yellow morphs of *P. delavayi*. Bumblebees with similar color receptors exhibit identical color recognition to honeybees. It has been demonstrated that syrphid flies can discern subtle color differences, but visual cues play a relatively minor role in the foraging behavior of many Diptera species (Huang et al., 2024). Syrphidae insects typically favor yellow and white flowers (Lunau et al., 2018) and are less responsive to other colors (Li et al., 2019). Observations confirm that these flies frequently visit yellow flowers, while their visits to red morphs are significantly less common. Moreover, The fluorescent properties often exhibited by pollen may also change how flower colors are perceived (Castellanos et al., 2006). The anther of *P. delavayi* emits blue fluorescence from its epidermis of the anther wall under UV light. Therefore, the manner in which pollinators perceive and distinguish between flowers with distinct colors, and how they respond to these perceived differences by preferred visits, may drive the divergence of flower colors among morphs or species (Trunschke et al., 2021).

Scent is a more important guide for pollinators in locating flowers that are not easily visible (Shuttleworth and Johnson, 2012). Terpenoids, which are among the most prominent plant volatiles, help mediate interactions between plants and pollinators (Schiestl, 2005; Schiestl and Schlüter, 2009; Li et al., 2023). Insects possess a highly sophisticated olfactory system, enabling them to detect and identify floral cues in the air, thereby responding and facilitating pollination driven by the volatiles emitted by the flowers (Zhang et al., 2023). Earlier studies have shown that certain compounds, such as linalool, act as innate attractants, eliciting strong responses in the antennae of bees and bumblebees (Kubo and Ono, 2014; Li et al., 2023). Notably, some studies have addressed whether increased concentrations of linalool result in stronger insect attraction. for instance, stingless bees display a significant tendency to prefer citrus, which comprises 12.7% linalool, over lemon, which is rich in 62.89% limonene, during feeding (Grajales-Conesa et al., 2012). Furthermore, the odours, which contain linalool, α-copaene, caryophyllene, terpinolene and β-cubebene, emit a fruity scent that may attract fly pollinators (Jaleel et al., 2019; Zhang et al., 2023; Huang et al., 2024). Our study revealed that that *P. delavayi* is a typical bee-pollinated flower (Wilson and Stine, 1996; Willmer, 2012; Li et al., 2023), containing abundant terpenoids in its petals and anthers. Floral volatiles vary in both the petals and anthers of the two flower morphs. Specifically, the red-flowered form of *P. delavayi* exhibits a diverse array of terpenoids, including linalool, α-cubebene, β-cubebene, caryophyllene and terpinolene, which play a crucial role in attracting bee and fly pollinators. Notably, the concentration of linalool is significantly higher in both the petals and anthers of red-flowered plants compared to those with yellow flowers.

Polliation is crucial for the sexual reproduction of seed plants, and the frequency of pollinator visits seems to be an effective predictor of reproductive success (Tewksbury et al., 2002). Pollinator groups vary in their perception, detection, and preferences for flower color, scent, shape, size, and rewards, which may lead to significant differences in fruit or seed set among floral morphologies (Pisanty et al., 2016). There were no differences in natural seed set between the yellow and red morphs of *P. delavay*i; however, pollinators showed a preference for yellow morphs, which demonstrated a male fitness advantage (Stanton, 1987). Our results support the view that higher pollinator visitation rates lead to increased seed set (Chen and Zuo, 2019). The generalized linear model (GLM) demonstrated that the frequency of visits by honeybees and bumblebees positively influenced seed production in naturally pollinated flowers, highlighting their crucial role as pollinators for the two morphs of *P. delavayi*. This finding is supported by prior research indicating that honeybees and bumblebees are primary pollinator assemblages, as evidenced by their high visitation frequency (Zych et al., 2013). In contrast, this research did not find evidence that increased visitation by syrphid flies significantly affects the reproductive success of *P. delavayi*. Reproductive success in many plants hinges on pollinators. However, environmental factors, such as temperature and altitude, can disrupt pollinator numbers and activity (Shrestha et al., 2014; Basnett et al., 2019). In high-altitude environments, the harsh environment (Li et al., 2016) and limited pollinator availability (Tur et al., 2016) may adversely affect reproductive success of *P. delavayi*. Harsh environmental conditions commonly result in diminished diversity and population of pollinators. These demographic changes can negatively influence the reproductive success of plants. In regions with restricted pollinator presence, plant reproductive success is to a large extent hindered, mainly due to pollen or pollinator limitation (Larson and Barrett, 2000). The setting rate of *P. delavayi* in XGLL is lower than that in LWS, highlighting the need for future studies on the effects of altitude gradients on pollinator-mediated interactions.

## 5 Conclusions

This study investigated the influence of floral traits on reproduction in two color morphs of *P. delavayi*, emphasizing their interaction with pollinating insects. Our findings indicate no significant difference in seed yields and seed sets between the two color morphs despite sharing pollinators and exhibiting minimal differentiation in floral morphological traits. However, there is variation in how pollinators perceive their flower colors. On the one hand, the yellow morphs contrast against the leaves background, enhancing their attractiveness to bees and flies. On the other hand, the red-flowered morph compensates for its visual disadvantage through olfactory cues, ensuring successful reproduction despite its lower visual attractiveness.

## Data Availability

The original contributions presented in the study are included in the article/**Supplementary Material**. Further inquiries can be directed to the corresponding author.
