# Supplementary material for: Reproductive strategies of two color morphs of Paeonia delavayi
Source: Front Plant Sci. 2025 Mar 19;16:1531186. doi: 10.3389/fpls.2025.1531186 (PMC11961923; doi:10.3389/fpls.2025.1531186)
Supplement: Supplementary file 1 [file DataSheet1.pdf]

Supplementary Material

Supplementary Table 1. Basic information on the studied sites of *P. delavayi*

Study Site	Abbreviation	Locality	Elevation (m)	Longitude (E)	Latitude (N)	Floral colour	Anther colour
Lianwang Mountain	LWS	C Yunnan province, China	3356	99.64	27.90	red	red
Shangri-La	XGLL	NW Yunnan province, China	2365	102.92	24.84	yellow	yellow

Supplementary Table 2. Effects of different pollination treatments (mean ± s.e.) among the two morphs of *P. delavayi* in XGLL(n = 23)

Parameters	Treatment									
	Yellow morphs	Red morphs								
	Natural pollination	Natural pollination	Emasculation	Artificial homogamy	Artificial geitonogamy	Artificial xenogamy	Anemophilous pollination	F	df	P
Seed yields	2.26 ±0.32	2.04 ±0.47[ab]	0.96 ±0.32[bc]	0.43 ±0.21[c]	0.83 ±0.3[c]	2.27 ±0.46[a]	0.09 ±0.06[c]	5.448	5	< 0.001
Fruit set (%)	14.25 ±1.9	10.58 ±2.52[ab]	5.04 ±1.63[bc]	2.33 ±1.11[c]	3.63 ±1.25[c]	12.82 ±2.77[a]	0.40 ±0.28[c]	5.328	5	< 0.001

Values (mean ± s.e.) with different letters in the same row indicate significant differences between treatments according to Tukey's tests at $p < 0.05$

Supplementary Table 3. The species and behavior of insects visiting *P. delavayi*

Study Site	Superfamily	Family	Species	Observed Number		single Flower-visiting time/s	visiting behavior	Rewards
				Red morphs	yellow morphs			
LWS	*Hymenoptera*	*Halictidae*	Halictidae sp.	10	12	75.67±72.62	Fly	pollen, Nectar
		Apeidae	Apis sp.	650	1307	35.45±22.40	Fly, Crawl	pollen, Nectar
			Bombus (Alpigenobombus) genalis	240	262	85.15±73.54	Fly, Crawl	pollen, Nectar
		Formicidae	*Formica fusca* Linnaeus	1536	929	284.06±177.57	Crawl	Predation, nectar
			Formica sinensis Wheeler	1626	813	296.14±181.43	Crawl	Predation, nectar
	Diptera	*Syrphidae*	Syrphidae sp.	46	118	178.47±167.64	Fly	pollen, Nectar
	Hemiptera	*Pyrrhocoridae*	*Lygaeus vicarius*	11	3	87.85±72.66	Crawl	Predation
			Dysdercus sp.	6	1	91.14±69.33	Crawl	Predation
		Lygaeidae	*Nysius ericae*	5	1	161.35±42.35	Crawl	Predation
XGLL	*Hymenoptera*	*Apeidae*	*Apis* sp.	221	285	33.81±27.56	Fly, Crawl	pollen, Nectar
		Formicidae	*Lasius himalayanus* Bingham	66	63	214.07±195.46	Crawl	Predation, nectar
	Diptera	*Muscidae*	*Musca domestica*	12	28	102.27±99.72	Fly	Nectar
		Syrphidae	Syrphidae sp.	71	164	156.89±148.41	Fly	pollen, Nectar
	Hemiptera	*Pyrrhocoridae*	*Lygaeus vicarius*	12	2	89.58±69.87	Crawl	Predation
			Dysdercus sp.	6	2	93.19±70.24	Crawl	Predation
		Lygaeidae	*Nysius ericae*	8	3	159.75±41.37	Crawl	Predation

Supplementary Table 4. Visitation frequency and residence time(s) (mean ± s.e.) by major pollinators of *P. delavayi* in XGLL

morphs	Insect pollinator	*Apis* sp.	*Syrphidae* sp.
Red Morph	Visitation frequency (visits/flower/h)	0.74±0.07[a]	0.24±0.04[a]
Yellow Morph		0.95±0.12[a]	0.45±0.07[b]
Red Morph	Residence time(s) (flower/h)	25.27±3.69[a]	28.81±6.48[a]
Yellow Morph		37.61±6.32[a]	66.14±11.9[b]

Values (mean ± s.e.) with different letters in the same column indicate significant differences according to Mann–Whitney U tests at $p < 0.05$.

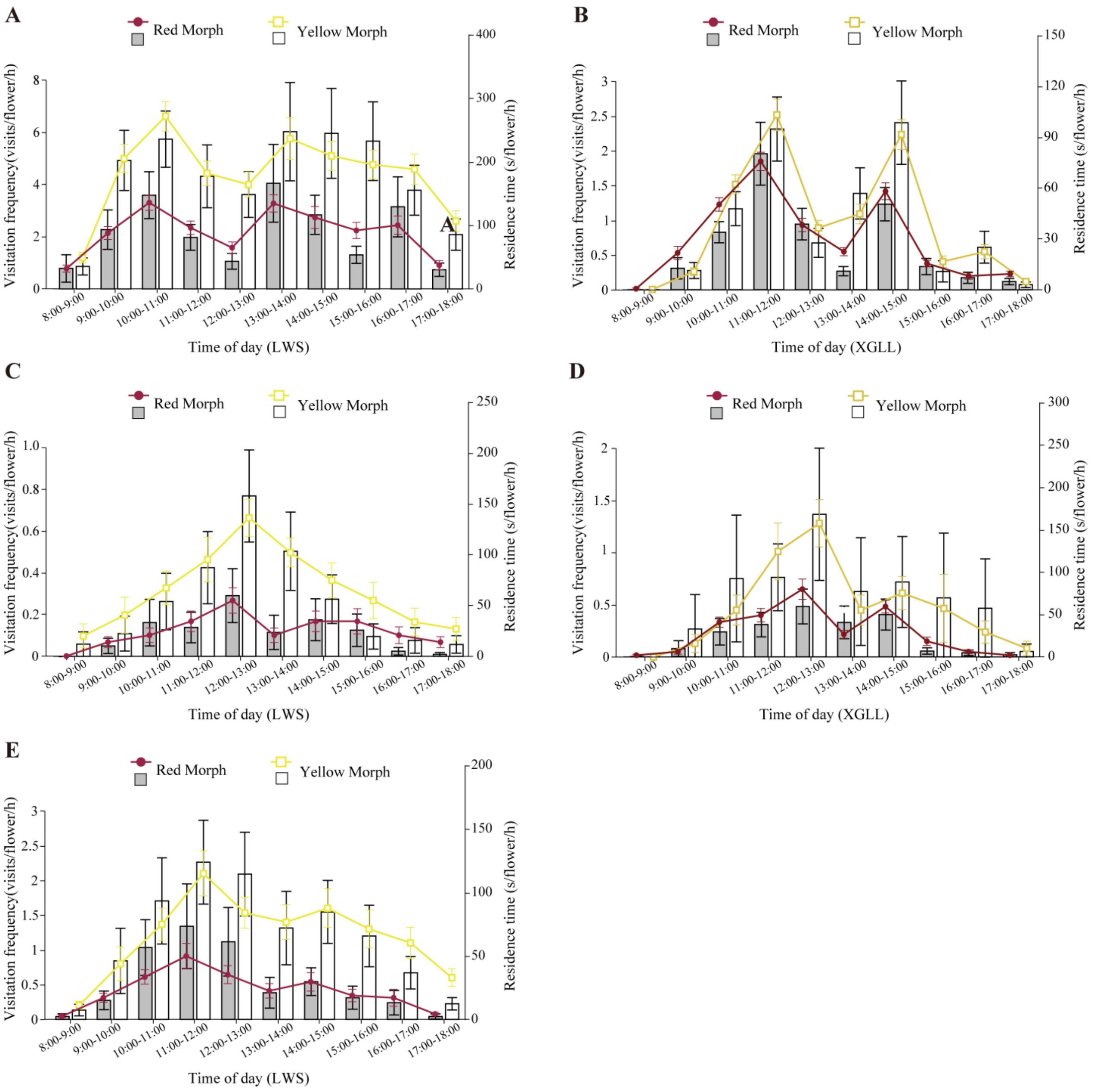

Supplementary Figure 1. Daily activity patterns (mean ±s.e.) of honeybees(A, B), syrphid flies (C, D) and bumblebees (E) in LWS and XGLL.

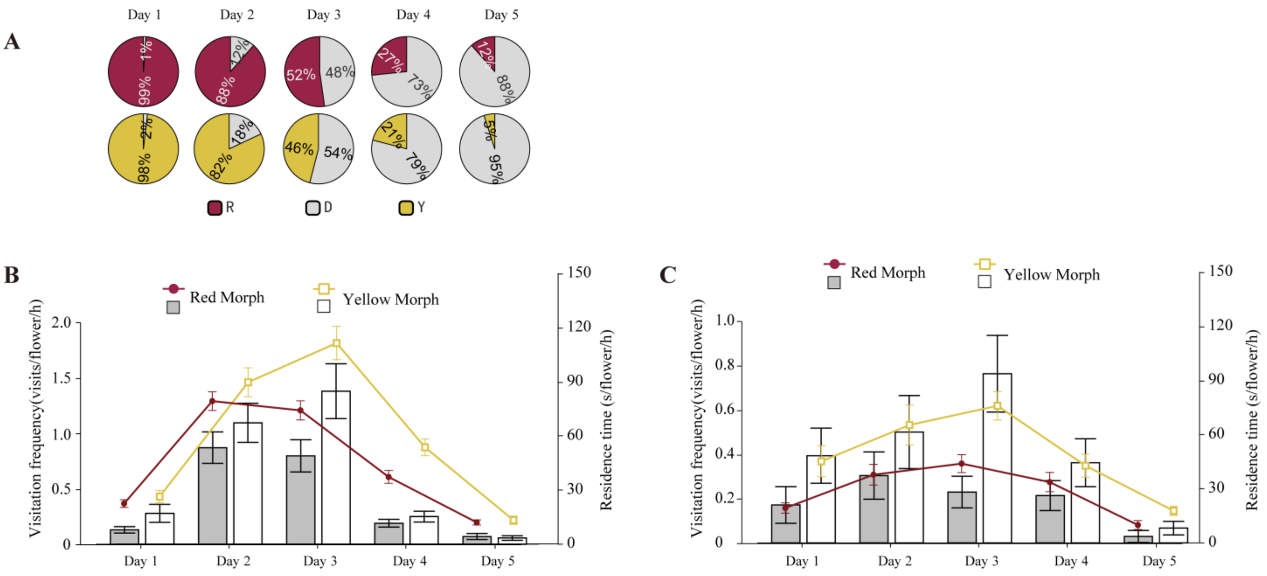

Supplementary Figure 2. Visitation frequency and residence time(s) (mean ±s.e.) of honeybees(B), syrphid flies (C) in XGLL, across different anther dehiscence schedules(A). R represents red indehiscent anthers, Y represents yellow indehiscent anthers, and D represents dehiscent anthers.

Supplementary Table 5. Results of linear regression analysis testing the response of the seed set of *P. delavayi* in each flower in average frequency of insect visits.

	Estimate	Std. Error	z value	Pr(>|t|)
(Intercept)	10.361	4.959	2.089	0.0463 *
honeybees	6.808	2.612	2.606	0.0147 *
Bumblebee	14.413	6.302	2.287	0.0303 *
Syrphid flie	4.769	5.005	0.953	0.3491

*： P < 0.05

Supplementary Table 6. Results of linear regression analysis testing the response of the seed set of *P. delavayi* in each flower in residence time(s) of insect visits.

	Estimate	Std. Error	t value	Pr(>|t|)
(Intercept)	12.13856	3.99337	3.04	0.00521 **
honeybees	0.16134	0.04711	3.424	0.00198 **
Bumblebee	0.2512	0.08351	3.008	0.00563 **
Syrphid flie	0.0184	0.03405	0.54	0.59337

**：P < 0.01

Supplementary Table 7. The floral morphological characteristics in *P. delavayi*, analysed by one-sample t-test.(mean ± S.E., N=60, mm).

Study Site	Floral characters	Flower diameter	Flower height	Stamen diameter	Stamen height	Pistil height	Stamen-pistil shortest	Stamen number	Petal number
LWS	red	46.65±0.76[a]	19.63±0.47[a]	20.18±0.37[a]	10.71±0.14[a]	11.82±0.27[a]	-1.11±0.32[a]	154.93±1.31[a]	8.92±0.10[a]
	yellow	44.63±0.58[b]	17.88±0.37[b]	18.80±0.22[b]	9.90±0.15[b]	9.71±0.14[b]	0.20±0.22[b]	125.92±1.88[b]	8.27±0.09[b]
	P	0.037	0.004	0.002	< 0.001	< 0.001	0.001	< 0.001	< 0.001
XGLL	red	55.59±1.15[a]	33.23±0.31[a]	28.37±0.31[a]	16.42±0.24[a]	14.83±0.32[a]	1.60±0.33[a]	260.53±6.57[a]	12.37±0.27[a]
	yellow	51.14±1.53[b]	31.01±0.37[b]	25.43±0.40[b]	13.92±0.28[b]	13.59±0.36[b]	1.32±0.36[b]	159.67±2.90[b]	8.82±0.09[b]
	P	0.022	< 0.001	< 0.001	< 0.001	0.012	0.009	< 0.001	< 0.001

Within the column, distinct letters denote significant differences among the two morphs for identical floral traits, as determined by One-way ANOVA at the 0.05 level.

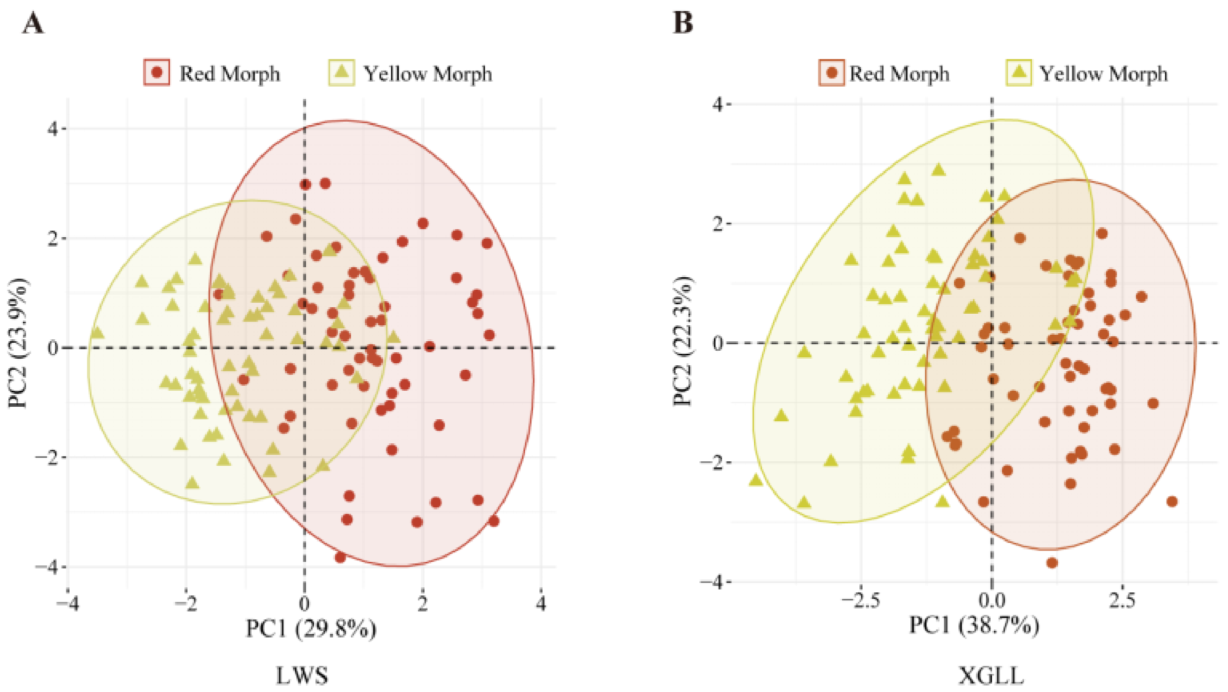

Supplementary Figure 3. Floral traits of two color morphs of *P. delavayi* analysed by PCA

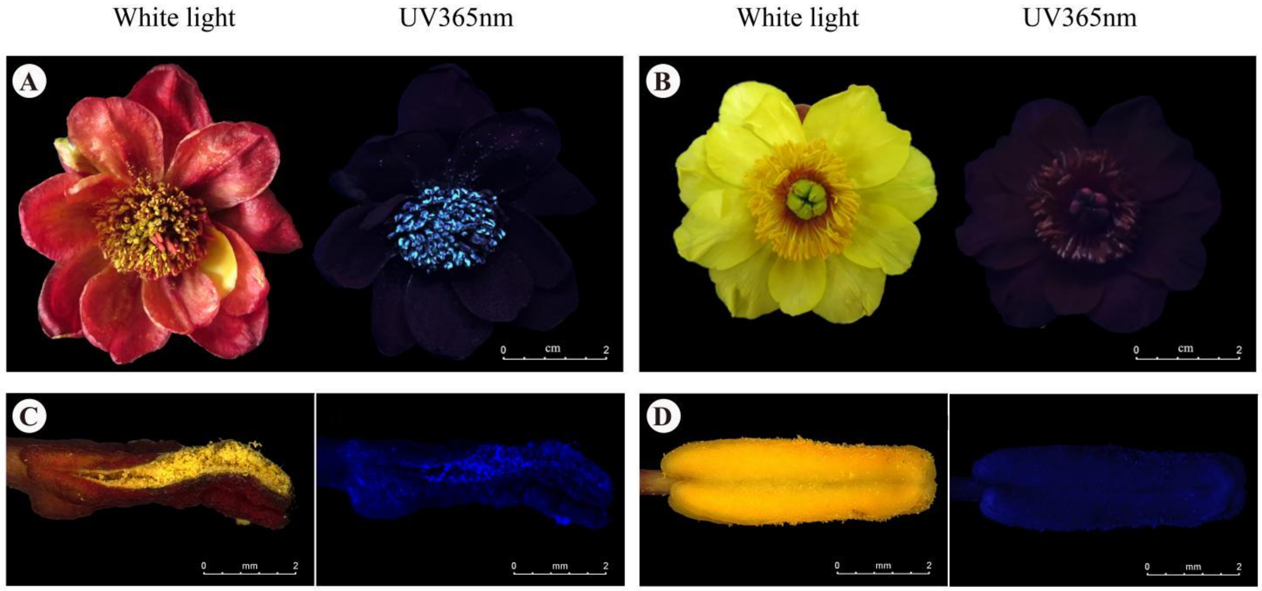

Supplementary Figure 4. Photographs of the flowers and anthers in XGLL. (A–B) Flower of *P. delavayi* and (C–D) its anther under white light (left) and UV 365 nm light (right). Scale bars, 2 mm for A–D.

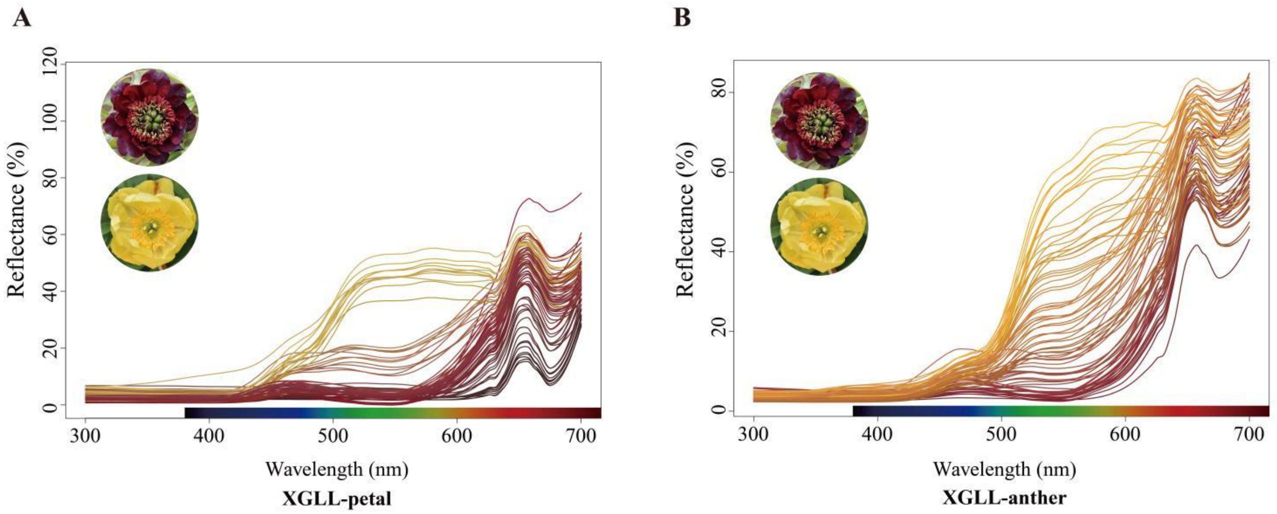

Supplementary Figure 5. Diffuse reflectance spectra of *P. delavayi* flowers. (A) Reflectances of petals and (B) its anthers in XGLL.

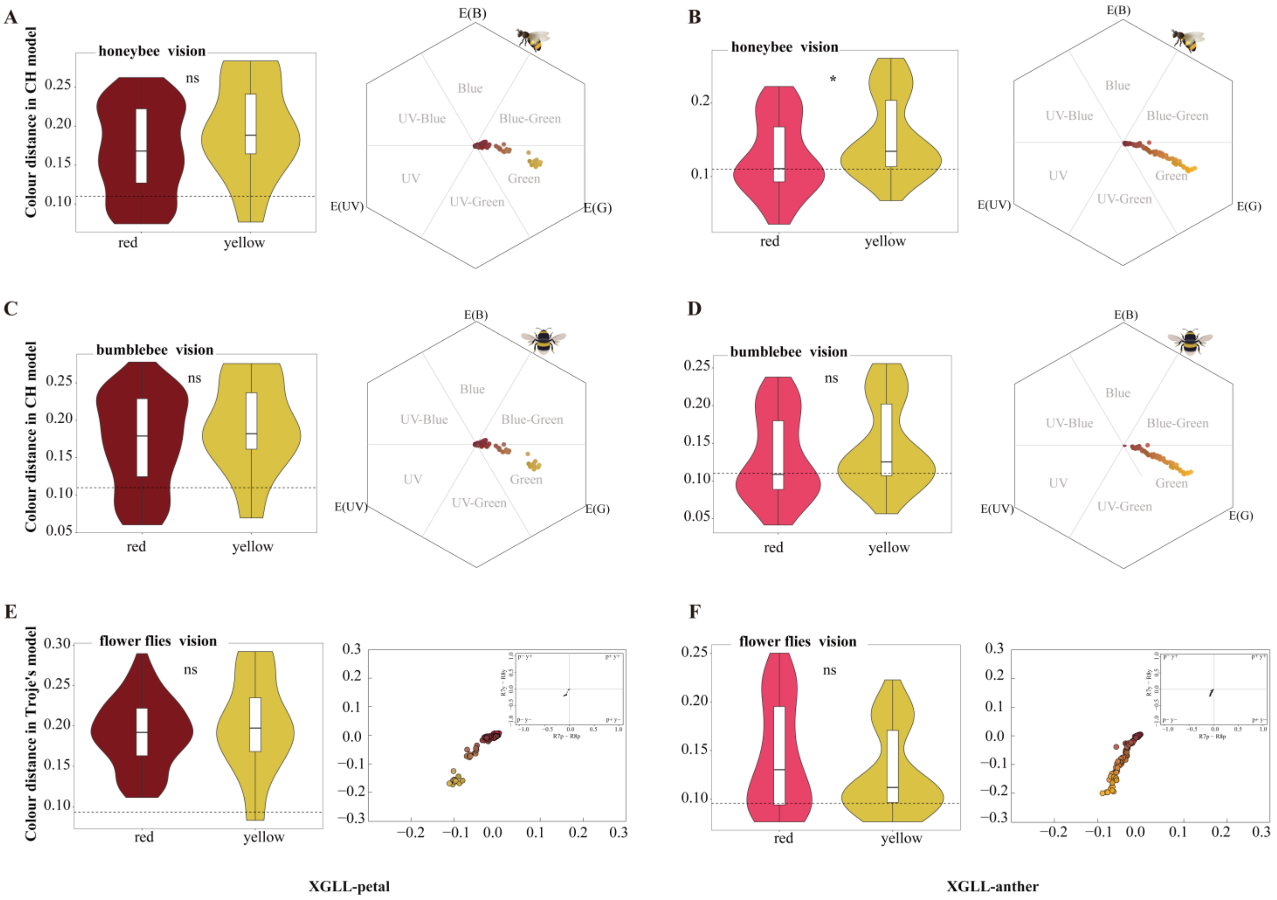

Supplementary Figure 6. Flower characteristics and color loci in pollinator color spaces across populations. (A–B) Colour distances between petals and leaves, as well as anthers and leaves, calculated using the bee color hexagon model. The dashed line indicates 0.11 hexagon units, which is

the discrimination threshold of bees. (C–D) Color distances between petals and leaves, as well as anthers and leaves, calculated using the bumble bee color hexagon model. The dashed line indicates 0.11 hexagon units, which is the discrimination threshold of bumblebees. (E–F) Color distances between petals and leaves , as well as anthers and leaves, calculated in the fly color model. The dashed line indicates the fly threshold of 0.096 Troje units. *p < 0.05; ns, not significant.

Supplementary Table 8. Mean relative amounts (%) of floral scent volatiles of *P. delavayi* in LWS (Mean ± S.E.).

**Compounds**	**red morphs**		**yellow morphs**	
	**petal**	**anther**	**petal**	**anther**
Samples of floral scents from different individuals	(n = 18)	(n = 18)	(n = 7)	(n = 7)
Number of compounds	87	85	49	53
Terpenoids (60)	43	29	25	20
3-Methylstyrene	0.16±0.01	—	—	—
α-muurolene	0.38±0.02	—	—	—
Isolongifolene	0.56±0.05	—	—	—
Caryophyllene Oxide	0.19±0.01	—	0.24±0.01	0.24±0.01
(-)-Isocaryophyllene	0.17±0.01	—	—	—
Myrcene	0.2±0.01	—	—	—
β-cubebene	6.99±0.64	1.19±0.23	4.15±0.06	2.51±0.11
DL-Limonene	0.12±0.01	0.05±0.03	—	—
[1S,2R,6R,7R,8S,(+)]-1,3-Dimethyl-8-(1-	—	—	0.1±0.01	—
(+)-Calarene	—	—	6.56±1.36	—
(-)-Alpha-Cubebene	0.41±0.01	0.6±0.01	0.78±0.06	1.14±0.06
2,2,6,6-tetramethylbicyclo[3.1.0]hex-3-ene	—	0.49±0.07	—	—
Tridecane, 3-methylene-	—	0.21±0.08	—	—
α-calacorene	—	—	0.09±0.01	—
Cyperene	0.28±0.04	—	—	—
Germacrene D	16.22±0.45	10.62±0.3	53.32±0.29	30.71±1.15
α-Cadinene	0.19±0.01	—	0.4±0.01	0.46±0.03
(-)-Alloaromadendrene	0.47±0.01	0.28±0.02	1.04±0.1	0.75±0.05
(+)-Aristol-9-ene	—	0.35±0.08	—	—
Artemisia triene	—	0.07±0.04	—	—
cadinadiene,cadina-1,4-diene	2.04±0.17	—	—	0.1±0.06
Bicycloelemene	0.65±0.02	—	—	0.52±0.07
(Z)-3,7-dimethylocta-1,3,6,-triene	0.19±0.06	—	—	—
(E)-3,7-dimethylocta-1,3,6-triene	0.99±0.04	1.03±0.08	—	—

(-)-Alpha-Copaene	0.41±0.01	0.39±0.02	0.72±0.05	1.22±0.17
C15 H24, Naphthalene, 1,2,3,4,4a,5,6,8a-octahydro-7-methyl-4-	0.37±0.03	0.52±0.02	1.54±0.04	1.16±0.06
2,5,6-Trimethyl-1,3,6-heptatriene	—	0.21±0.07	—	—
1-(1,4-dimethylcyclohex-3-en-1-yl)ethan-1-one	—	0.31±0.08	—	—
(-)-Alpha-Cedrene	—	—	0.09±0.03	—
(-)-Thujopsen	0.5±0.04	—	—	—
(+)-Delta-Cadinene	2.2±0.04	1.03±0.02	4.04±0.05	2.32±0.09
beta-maaliene	0.19±0.01	—	—	—
(+)-Aromadendrene	0.21±0.02	0.34±0.23	0.52±0.08	0.79±0.09
(1R)-(+)-Trans-Isolimonene	—	—	0.04±0.02	—
beta-patchoulene	—	0.56±0.08	0.15±0.01	0.1±0.03
1,5,5-Trimethyl-6-methylenecyclohexene	0.3±0.01	0.44±0.01	0.88±0.02	—
Beta-Elemene	0.5±0.07	—	—	—
α-Bourbonene	—	—	—	0.05±0.03
β-bourbonene	—	0.08±0.02	0.04±0.03	—
beta-Cadinene	1.33±0.07	0.13±0.04	0.11±0.07	—
α-Patchoulene	2±0.41	—	—	—
Terpinolene	—	0.63±0.04	—	—
(+)-Alpha-Longipinene	0.51±0.02	—	—	—
(S)-(-)-Limonene	0.15±0.04	—	—	—
2-Methylstyrene	0.06±0.03	—	—	—
3,3-Dimethyl-2-(2,7-octadienyl)-1-cyclobutene	0.09±0.03	—	—	—
Alpha-Caryophyllene	1.88±0.02	1.09±0.03	3.19±0.02	2.37±0.1
(6Z)-6-[(Z)-2-Butenylidene]-1,5,5-trimethyl-1-cyclohexene	—	—	—	0.19±0.2
(+)-Beta-Funebrene	—	0.37±0.04	—	—
Camphene	0.34±0.01	—	—	—
alpha-Pinene	0.23±0.01	—	—	—
Beta-Caryophyllene	3.85±0.05	2.45±0.05	6.37±0.09	5.05±0.21
3-Methyl-1,4,6-Heptatriene	0.05±0.03	—	—	—
(-)-Isoledene	0.3±0.03	—	—	—
Linalool	15.02±0.31	4.3±0.18	0.79±0.28	0.14±0.03
T-muurolol	0.45±0.01	0.06±0.03	1.24±0.09	0.35±0.04
tetrahydro-alpha,alpha,5-trimethyl-5-vinylfuran-2-methanol	4.94±0.75	5±0.27	0.8±0.24	—
α-eudesmol	0.75±0.06	—	—	—
α-cadinol	0.92±0.12	0.18±0.03	1.28±0.04	0.46±0.05
cis-alpha,alpha,5-trimethyl-5-vinyltetrahydrofuran-2-methanol	17.04±0.5	1.02±0.03	—	—
Alcohols (15)	8	6	7	6
Benzyl alcohol	—	0.53±0.01	—	0.23±0.04
Cinnamyl alcohol	6.35±0.27	0.27±0.03	—	—

(1R,4aβ)-Decahydro-1β-[(E)-5-hydroxy-3-methyl-3-pentenyl]-	—	—	0.23±0.06	—
2-((4aR)-1,2,3,4,4alpha,5,6,7-octahydro-4alpha,8-	0.16±0.02	—	—	—
3-Phenyl-1-propanol	0.86±0.02	—	—	—
2,2,6-Trimethyl-6-vinyltetrahydro-2h-pyran-3-ol	3.06±0.06	0.21±0.02	0.29±0.12	—
(R)-(+)-1-Phenylethanol	—	1.67±0.14	0.33±0.07	—
Bicyclo[3.1.0]hexan-2-ol	—	—	—	0.07±0.04
beta-Eudesmol	0.13±0.01	—	—	—
[1aR-(1aalpha,4beta,4abeta,7alpha,7abeta,7balpha)]-decahydro-	—	—	0.32±0.02	—
T-cadinol	0.64±0.01	—	—	0.19±0.11
Phenethyl alcohol	—	—	0.41±0.05	0.06±0.04
alpha-elemol	0.71±0.02	0.17±0.01	0.71±0.05	0.11±0.03
4,8-dimethylnona-3,7-dien-2-ol	—	—	0.12±0.04	—
DL-1-Phenethylalcohol	0.42±0.02	0.09±0.03	—	0.57±0.06
Esters (23)	10	18	3	8
Methyl cinnamate	—	—	—	0.4±0.04
Cinnamyl acetate	0.62±0.03	—	—	—
Ethyl caprate	13.79±0.66	52.34±1.33	11.67±0.5	42.35±2.32
Methyl decanoate	0.27±0.04	0.82±0.03	—	0.3±0.04
Ethyl 2-hydroxybenzoate	—	0.23±0.03	—	—
Methyl salicylate	—	0.13±0.04	—	—
1-Phenylethyl propionate	0.14±0.02	0.37±0.11	—	—
Cinnamylcetate	0.5±0.06	—	—	—
N-Amyl isovalerate	—	0.18±0.11	—	—
Butanoic acid,2-methyl-, 3-methylbutyl ester	—	0.11±0.07	—	—
FEMA 2686	0.05±0.03	—	—	—
Benzoic acid, 2,5-bis[(trimethylsilyl)oxy]-, trimethylsilyl ester	0.21±0.03	—	—	—
palmitic acid benzyl ester	—	0.09±0.05	—	—
alpha-methylbenzyl acetate	—	0.16±0.05	—	—
Methyl isovalerate	—	1.11±0.11	—	—
(-)-Bornyl acetate	0.4±0.03	0.6±0.06	0.03±0.02	0.24±0.04
Ethyl nicotinate	—	0.44±0.08	—	—
Isoamyl isovalerate	—	0.19±0.11	—	—
Diethyl phthalate	0.12±0.01	0.07±0.06	0.31±0.06	0.26±0.03
Diisobutyl phthalate	0.5±0.02	0.21±0.06	—	—
Methyl benzoate	—	0.45±0.02	—	0.11±0.06
Methyl nicotinate	—	0.63±0.04	—	0.04±0.03
Ethyl benzoate	—	3.66±0.46	—	0.34±0.2
Aromatics (8)	4	4	3	3

1,3-Divinylbenzene	—	0.33±0.03	—	—
Divinylbenzene	0.26±0.02	0.34±0.01	—	—
1,2,3,4,6,8alpha-Hexahydro-1-isopropyl-4,7-dimethylnaphthalene	0.12±0.01	0.04±0.02	0.24±0.23	0.17±0.01
1,2,4aβ,5,6,8aβ-Hexahydro-4,7-dimethyl-1β-	—	—	—	0.28±0.04
triisopropylbenzene	—	0.49±0.04	—	0.16±0.05
1-isopropyl-7-methyl-4-methylene-1,2,3,4,4a,5,6,8a-	0.28±0.08	—	1.04±0.01	—
1,1,6-Trimethyl-1,2-dihydronaphthalene	—	—	0.03±0.02	—
1-(2-methylprop-2-enoxy)-4-tert-butyl-benzene	2.17±0.08	—	—	—
Alkanes (22)	11	10	0	6
2,7-Dimethyloctane	0.05±0.03	—	—	—
Triallylsilane	—	0.26±0.02	—	—
Dodecane	—	0.08±0.02	—	—
Dodecamethylpentasiloxane	—	0.5±0.03	—	—
6,6-Dimethyl-3-methylenebicyclo[3.1.1]heptane	0.13±0.04	—	—	—
Dodecane,3-methyl-	—	0.24±0.01	—	—
3,4-Dimethylundecane	0.06±0.03	—	—	—
Decylcyclopentane	—	0.17±0.05	—	—
2,6,11-Trimethyldodecane	—	—	—	0.13±0.04
1-methyl-4-(1-methylethylidene)-2-(1-methylvinyl)-1-	0.9±0.09	0.54±0.04	—	—
2,6,10-Trimethylpentadecane	—	—	—	0.19±0.06
Dodecamethylcyclohexasiloxane	0.14±0.03	0.4±0.02	—	0.38±0.02
Decamethylcyclopentasiloxane	—	—	—	0.1±0.03
N-Hexadecane-d34	—	—	—	0.19±0.02
5-propyltridecane	0.09±0.05	—	—	—
2,3-Diphenylbutane	0.07±0.04	—	—	—
Octadecane	0.13±0.04	—	—	—
1-Methoxyadamantane	0.05±0.03	—	—	—
N-Heptadecane	0.06±0.03	0.07±0.01	—	0.18±0.02
N-Heneicosane	—	0.09±0.03	—	—
3-Methyltridecane	—	0.41±0.04	—	—
1-Iodo-2-methylundecane	0.07±0.04	—	—	—
Ketones (4)	3	3	1	1
trans-5-methyl-2-(1-methylvinyl)cyclohexan-1-one	0.05±0.03	—	—	—
Fitone	0.12±0.02	0.15±0.02	—	—
Camphor	—	0.08±0.05	—	—
Acetophenone	0.82±0.05	3.05±0.1	2.32±0.83	1.48±0.28
Phenols (8)	0	5	2	2
2,4-Dimethylphenol	—	—	0.14±0.04	—
2-methoxy-3-(2-propenyl)，Phenol	—	19.63±0.58	—	—

2,6-Dimethyl-4-(methoxymethyl)phenol	—	—	0.17±0.05	—
3-Aminophenol	—	0.15±0.04	—	—
(E)-2-methoxy-4-(prop-1-enyl)phenol	—	0.23±0.07	—	—
Methyl eugenol	—	0.07±0.04	—	—
2,5-Dimethylphenol	—	—	—	0.05±0.03
Eugenol	—	5.42±0.38	—	0.2±0.04
***Aldehyde*(1)**	1	0	0	0
Cinnamaldehyde	0.23±0.01	—	—	—
Others (20)	7	10	8	7
Bicyclo[8.1.0]undeca-2,6-diene, 3,7,11,11-tetramethyl-,	1.59±0.05	0.82±0.02	3.79±0.09	2.77±0.17
N-Methyl-β,3,4-tris(trimethylsiloxy)benzeneethanamine	—	0.18±0.05	—	—
2-Acetyl pyrrole	—	0.05±0.03	—	0.12±0.07
3,4,4a,5,6,7,8,8a-Octahydro-5-methylene-8-vinyl-2-naphthoic	—	—	0.06±0.03	—
Triallylphosphine	—	0.18±0.1	—	—
Nonane,3,7-dimethyl-	—	0.33±0.1	—	—
(14α)-7,8-Didehydro-3-methoxy-17-methyl-6-	—	0.6±0.03	—	—
1,4-Methanoazulen-7(1H)-one, octahydro-4,8,8,9-tetramethyl-,	—	—	0.13±0.05	—
Dendrolasin	—	—	0.12±0.02	—
1α-Isopropyl-4,7-dimethyl-1,2,4aβ,5,6,8aβ-hexahydronaphthalene	0.74±0.02	0.53±0.02	1.94±0.14	0.91±0.08
Boldine dimethyl ether		—	—	0.12±0.04
1,2,4a,5,6,8a-hexahydro-1-isopropyl-4,7-dimethylnaphthalene	0.27±0.08	—	—	0.19±0.11
(-)-Alpha-gurjunene	0.03±0.02	—	0.07±0.02	0.11±0.03
Cyclohexaneethanol, 2-methylene-	0.05±0.03	—	—	—
(1R)-1α-Methyl-4β-isopropyl-6-methylene-1,2,3,4,6,7,8,8aβ-	1.09±0.07	0.69±0.03	0.16±0.05	—
Bicyclo[3.2.0]hept-6-en-2-one, 6-propyl-	—	0.03±0.02	—	—
3,4-Diaminopyridine	—	0.09±0.05	—	—
1-butylpyrrole	—	—	0.04±0.02	—
Tricyclo[5.4.0.03,9]undecane, 1,3,6-trimethyl-2-methylene-,	0.1±0.02	—	—	—
9H-Pyrrolo[3',4':3,4]pyrrolo[2,1-a]phthalazine-9,11(10H)-dione,	—	—	—	0.15±0.04
